# New Intrinsic Ecological Mechanisms of Leaf Nutrient Resorption in Temperate Deciduous Trees

**DOI:** 10.3390/plants13121659

**Published:** 2024-06-15

**Authors:** Xingchang Wang, Yanmin Guo, Qi Wang, Jun Pan, Xiankui Quan, Jiacun Gu, Chuankuan Wang

**Affiliations:** 1 Center for Ecological Research, Northeast Forestry University, Harbin 150040, China; xcwang_cer@nefu.edu.cn (X.W.); guoym463954307@nefu.edu.cn (Y.G.); panjun@scbg.ac.cn (J.P.); quanxiankui@nefu.edu.cn (X.Q.); 2 Key Laboratory of Sustainable Forest Ecosystem Management, Ministry of Education, Northeast Forestry University, Harbin 150040, China; gjcnefu@nefu.edu.cn; 3 National-Regional Joint Engineering Research Center for Soil Pollution Control and Remediation in South China, Guangdong Key Laboratory of Integrated Agro-Environmental Pollution Control and Management, Institute of Eco-Environmental and Soil Sciences, Guangdong Academy of Science, Guangzhou 510650, China

**Keywords:** nitrogen resorption, phosphorous resorption, leaf senescence phenology, pigments, nonstructural carbohydrates, carbon sink saturation hypothesis

## Abstract

Leaf nutrient resorption is a critical process in plant nutrient conservation during leaf senescence. However, the ecological mechanisms underlying the large variability in nitrogen (NRE) and phosphorous (PRE) resorption efficiencies among trees remain poorly understood. We conducted a comprehensive study on NRE and PRE variability using 61 tree individuals of 10 temperate broad-leaved tree species. Three potentially interrelated intrinsic ecological mechanisms (i.e., leaf senescence phenology, leaf pigments, and energy residual) were verified. We found that a delayed leaf senescence date, increased degradation of chlorophylls and carotenoids, biosynthesis of anthocyanins, and reduced nonstructural carbohydrates were all positively correlated with NRE and PRE at the individual tree level. The intrinsic factors affecting resorption efficiency were ranked in decreasing order of importance: leaf pigments > energy residual > senescence phenology. These factors explained more variability in NRE than in PRE. Our findings highlight the significance of these three ecological mechanisms in leaf nutrient resorption and have important implications for understanding how nutrient resorption responds to climate change.

## 1. Introduction

Nitrogen (N) and phosphorus (P) are crucial nutrients that restrict primary productivity and carbon storage in terrestrial ecosystems [1]. To cope with nutrient limitation, plants transfer nutrients from senescing organs to living organs for future use, a process known as nutrient resorption [2]. Nutrient resorption prolongs the residence time of nutrients in plant tissues, mitigates the risk of nutrient loss during litter decomposition, thus playing a pivotal role in the carbon cycling of forest ecosystems [3]. One key metric of nutrient conservation strategies is nutrient resorption efficiency (NuRE), which denotes the proportion of nutrients translocated during leaf senescence [4,5]. The global mean N (NRE) and P (PRE) resorption efficiencies for woody plants were approximately 50% [6]; however, they exhibit considerable variability among sites, species, and individuals and years [7,8,9,10]. It is essential to explore the ecological mechanisms underlying the variation in NuRE for gaining a deeper understanding of nutrient strategies in forest tree species [2,7,11].

Several basic ecological mechanisms were successfully used to explain variations in leaf nutrient resorption among species or individual plants in previous studies. Green-leaf nutrient concentration [4,12], nutrient availability [13,14,15], relative nutrient limitation, and ecological stoichiometry [16,17,18] are widespread mechanisms of NRE and PRE. However, these mechanisms use nutrient levels themselves to interpret nutrient resorption, which are somewhat statistically dependent. The ecological mechanisms of nutrient resorption beyond nutrient concentration-based variables remain undefined. Nutrient allocation among structural, metabolic, and soluble fractions are important physiological mechanisms of nutrient resorption [2]. However, measurements of nutrient allocation fractions in green and senesced leaves are time-consuming. In contrast, some key, easily measured processes (i.e., the leaf abscission phenology, the degree of senescence indicated by chlorophylls, and the energy changes) are poorly understood in relation to N and P resorption [2]. Clearly, new ecological mechanisms are needed to explain, simulate, and predict leaf nutrient resorption variations across species and individual plants.

Autumn leaf abscission phenology is one of the potential ecological mechanisms responsible for variability in leaf nutrient resorption. The process of leaf senescence in autumn involves chlorophyll degradation, leaf color transformation, reduction in photosynthesis, nutrient resorption, and ultimately, leaf shedding [19]. Extending the duration of nutrient resorption by delaying leaf abscission can enhance nutrient resorption efficiency in non-marcescent trees [2]. Global climate warming may provide a longer period for resorption by delaying leaf senescence in temperate forest tree species [20,21,22], potentially benefiting nutrient resorption [23]. However, delayed autumn leaf senescence also increases the risk of frost damage, which can abruptly interrupt senescence and suppress the nutrient resorption process by causing leaf abscission [24]. Despite these uncertainties, the relationship between autumn leaf phenology and nutrient resorption efficiency remains poorly assessed. To address this gap, we will explore our first mechanism, the leaf senescence phenology hypothesis, which posits that delaying the leaf-fall date can increase nutrient resorption efficiency by reducing the nutrient remaining in senesced leaves.

Another factor contributing to the variability in leaf nutrient resorption is the changes in leaf pigments during senescence. The vibrant and colorful autumn leaves in temperate deciduous forests serve as a strong indicator of nutrient resorption [25,26,27]. The degradation of chlorophylls marks the onset of leaf senescence at the end of the growing season [28], leading to the appearance of other pigments such as remained carotenoids and newly synthesized anthocyanins, which impart a yellow-orange-red hue to the leaves [29]. The degradation of chloroplast pigments initiates a series of biochemical reactions necessary for the synthesis of secondary metabolites and releases simple metabolites containing nutrients [19,30,31,32], thereby facilitating nutrient translocation [30,33]. Newly synthesized, red-colored anthocyanins in some species may promote leaf nutrient resorption by providing photoprotection for the leaves [34] or serving as a symptom of nutrient deficiency (a negative correlation between NuRE and the corresponding nutrient concentraion in green leaves) [35,36,37]. Previous studies have found that species with higher red-colored leaves had higher NRE in comparison with green-color-leafed species, supporting the positive effect of anthocyanins on nutrient resorption [29,34,38]. However, when assessing the symptoms of nutrient deficiency, it is important to consider the possibility of a negative correlation between anthocyanins and nutrient concentration in green leaves [36]. Surprisingly, few studies have quantitatively evaluated the relationship of leaf pigments (degradation of chlorophylls and carotenoids and synthesis of anthocyanins) with NuRE, particularly the PRE. We propose the second ecological mechanism as the leaf pigments hypothesis: higher degradation of chloroplast pigments and increased synthesis of anthocyanins enhance NuRE. The third ecological mechanism underlying leaf nutrient resorption involves the balance of energy (carbon) supply and demand. Nutrient resorption is an active and energy-consuming process [2]. Nonstructural carbohydrates (NSCs), mainly composed of soluble sugars and starch, are widely used as measures of carbon balance in plants under various conditions [39,40]. Although NSCs can provide energy for resorption, the accumulation of leaf NSCs, particularly soluble sugars, can trigger leaf senescence [19,41]. Therefore, a high NSC concentration may reduce NuRE by shortening the resorption period. Additionally, NSCs after senescence can be considered as the afterlife energy residual and thus are expected to be negatively correlated with NuRE. Despite the potential key role of NSCs in leaf nutrient resorption [42,43], the relationship between NSC parameters and NuRE remains largely unknown. We aim to test the third hypothesis, known as the energy residual or carbon sink saturation hypothesis: higher NSC levels in both green and senesced leaves suppress NuRE.

The three new ecological mechanisms, beyond previous nutrient concentration-based interpretations, may be interrelated. They represent different aspects of leaf senescence, and leaf abscission is the final step of senescence [2], and is an apparent factor, while the latter two mechanisms are more physiological and directly related to leaf senescence. To validate the three hypotheses and assess the relative importance and interactive effects of intrinsic variables on nitrogen (N) and phosphorus (P) resorption, we conducted a comprehensive analysis of 61 trees from 10 deciduous broad-leaved species in a secondary forest. We measured the concentrations of chlorophylls, carotenoids, anthocyanins, N, P, and NSCs in both green and senesced leaves, as well as the autumn leaf-fall phenology, to investigate the intrinsic ecological mechanisms underlying the leaf NuRE variability. Our findings provide new perspectives on the conceptual understanding and quantitative modeling of leaf nutrient resorption in co-occurring temperate forest trees.

## 2. Results

### 2.1. Leaf Senescence Phenology

**Figure 1:**
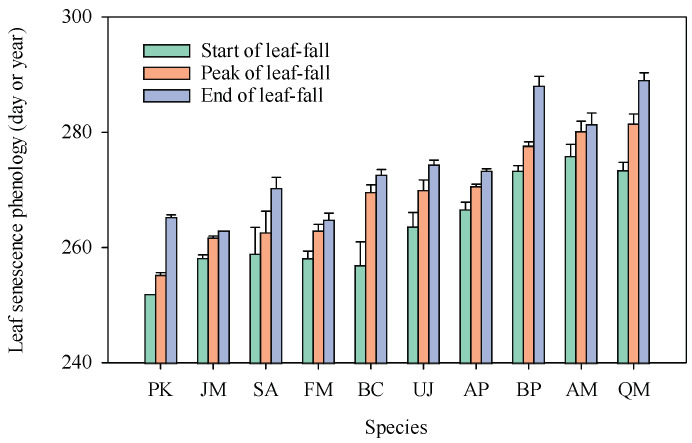
Leaf senescence phenological parameters. The leaves of each tree species are ranked in the order of DOY of peak of leaf fall. Species abbreviations: *Populus koreana* (PK), *Juglans mandshurica* (JM), *Syinga reticulata* var. *mandshurica* (SA), *Fraxinus mandshurica* (FM), *Betula costata* (BC), *Ulmus davidiana* var. *japonica* (UJ), *Acer pictum* subsp. *mono* (AP), *Betula platyphylla* (BP), *Acer mandshuricum* (AM), and *Quercus mongolica* (QM). The error bar is standard error. The same is below.

There were notable variations in leaf senescence phenology among the 10 tree species (Figure 1). The peak of leaf fall ranged between 255 and 290 days, indicating a difference of 35 days. *Populus koreana* displayed the earliest peak fall (DOY252 or 12 September), while *Quercus mongolica* exhibited the latest (DOY281 or 17 October). Moreover, there were significantly positive correlations between the start, the peak, and the end of leaf fall (Figure S1).

### 2.2. Changes in Leaf Pigments and Nonstructural Carbohydrates

**Figure 2:**
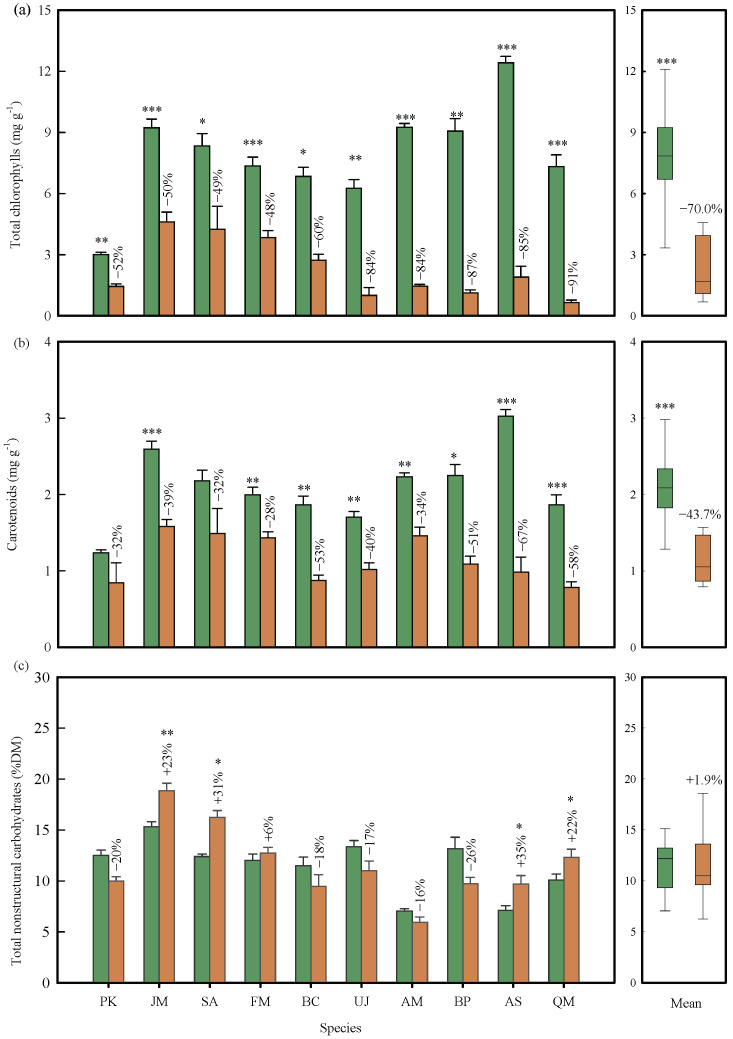
Comparison of the concentrations of total chlorophyll, carotenoids, and total nonstructural carbohydrates before and after leaf senescence. Total chlorophyll (**a**), carotenoids (**b**), and total nonstructural carbohydrates (**c**). Species abbreviations are given in Table 1. The green box indicates green leaves, and brown indicates senesced leaves, and the error bar is standard error. The percentage change is shown for each species, calculated from the mean change of each individual tree. The right figure is the mean of the 10 species. ***, **, * indicate significant at the 0.001, 0.01, and 0.05 levels, respectively.

Leaf color for most trees transitioned to yellow, brown, and red during senescence due to changes in leaf pigmentation (Figure S2). The levels of photosynthetic pigments notably decreased during leaf senescence (Figure 2a). The total chlorophyll concentration in the senesced leaves of the 10 tree species decreased by an average of 71% relative to the green leaves, with the species mean ranging from a 48% decrease (*F. mandshurica*) to 91% (*Q. mongolica*) (Figure 2a, *p* < 0.01). On average, the carotenoids concentration during senescence decreased by 46% (*p* < 0.01 by the paired *t*-test), with the maximum reduction (67%) in *A. mandshuricum* and the minimum reduction (28%) in *F. mandshurica*, respectively (Figure 2b). The changes in total chlorophylls and carotenoids based on leaf area (Figures S3 and S4) were consistent with that based on leaf mass. Anthocyanins were produced in all individuals of A. mandshurica, three of the nine individuals of *U. davidiana* var. *japonica*, and one of the three *A*. *pictum* subsp. *mono*, but not in the other species (Figure S5).

The TNC concentration showed no significant differences before and after leaf senescence when all species were combined (Figure 2c, *p* > 0.05). The changes in leaf TNC concentration ranged from –26% *(J. mandshurica*) to +35% (*Q. mongolica*) across the 10 tree species. Specifically, the leaf TNC concentrations of four species (*J. mandshurica*, S. *reticulata* var. *mandshurica*, *A. mandshuricum*, and *Q. mongolica*) significantly increased during the senescence period (*p* < 0.05), but not in the other species (*p* > 0.05). These mass-based results were consistent with the changes in TNC based on leaf area (Figure S6).

### 2.3. Correlations of Resorption Efficiency with Leaf Phenology, Pigments, and Nonstructural Carbohydrates

**Figure 3:**
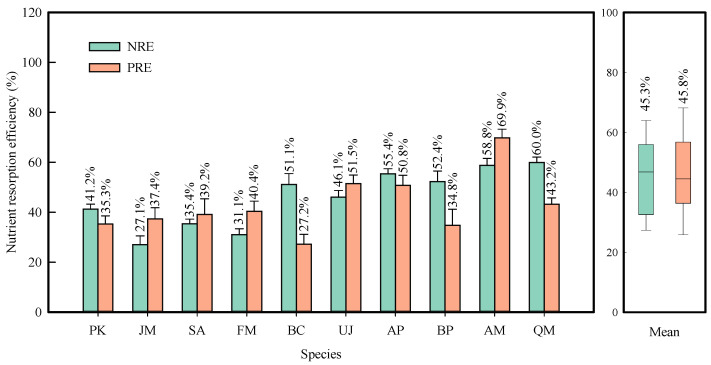
Nutrient resorption efficiency for the 10 tree species. NRE, nitrogen resorption efficiency; PRE, phosphorous resorption efficiency. Species abbreviations are given in Table 1.

The NRE and PRE ranged from 21.0% to 60.0% and from 27.2% to 77.9% across the 10 species, with mean values of 45.3% and 46.7%, respectively (Figure 3). As expected, leaf NuRE showed a significant correlation with leaf abscission phenology, photosynthetic pigments, and NSC concentrations (Figure S7). The NuRE increased as leaf fall delayed across the 61 tree individuals, with variations at different leaf fall stages, and the correlation was stronger for NRE (Figure S8). Senesced leaves with higher chlorophyll and carotenoid concentrations had lower NuRE (Figure S9). Additionally, anthocyanins concentration in senesced leaves was positively correlated with NuRE (Figure S10) and green-leaf N concentration (Figure S11) but not significantly correlated with green-leaf P concentration (Figure S11). Furthermore, NuRE decreased with the increase in NSC concentration in both green and senesced leaves (Figures S12 and S13), although it was not correlated with the change in NSCs (Table S2 and Figure S7). The species-mean data also supported these hypotheses, although some correlation coefficients decreased due to small sample size (61 versus10, Table S3).

### 2.4. Intrinsic Factor Apportionment of Resorption Efficiency

**Figure 4:**
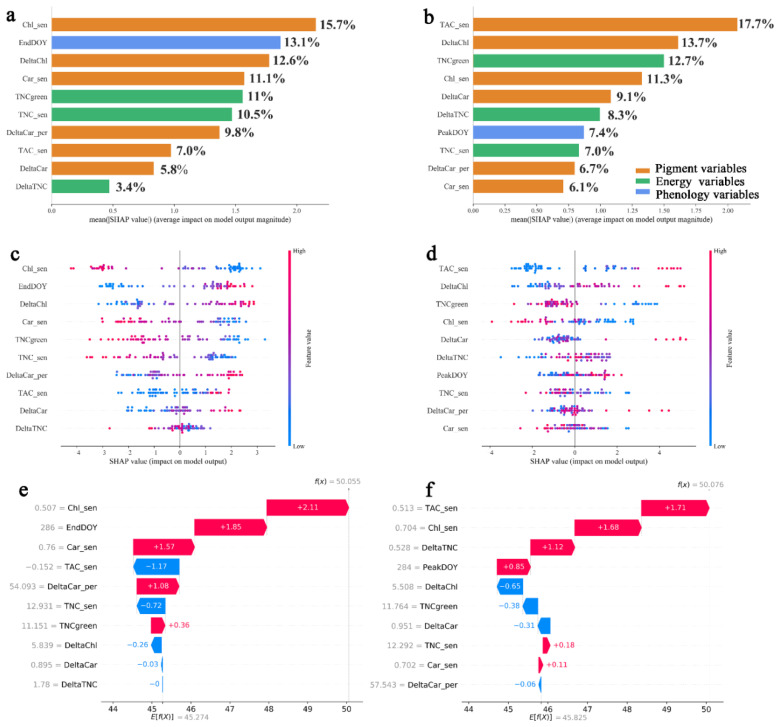
The influencing factor quantification and apportionment of the NRE and PRE from SHAP values. Bar plot of the mean absolute SHAP values of the influencing factors affecting the NRE (**a**) and PRE (**b**). A set of bee swarm plots, where each dot corresponds to an individual tree in the study, for the NRE (**c**) and PRE (**d**). The dot’s position on the *x*-axis shows the impact that feature has on the model’s prediction for that sample. When multiple dots land at the same x position, they pile up to show density. (**e**) Individual tree prediction for the NRE. (**f**) Individual tree prediction for the PRE. The red and blue colors are positive and negative feature values, respectively.

Both NRE and PRE are significantly influenced by the pigments, energy, and phenology variables (Figure 4), with the corresponding R^2^ values of 0.85 and 0.60 in the random forest model. When considering the entire dataset, the factor apportionment indicates a decreasing order for both NRE and PRE: pigment > energy > phenology. Specifically, the pigments, energy, and phenology variables contributed 62.0%, 24.9%, and 13.1% to the NRE (Figure 4a), respectively. These variables accounted for 64.6%, 28.0%, and 7.4% of the PRE, respectively (Figure 4b). The top three most important influencing factors of the NRE were Chl_sen, EndDOY, and DeltaChl, with relative importance of 15.7%, 13.1%, and 12.6%, respectively (Figure 4a). A negative SHAP value indicates a negative impact of the factors on the NRE. High Chl_sen reduced the NRE, while later EndDOY and higher DeltaChl increased the NRE. In contrast, high Car_sen, TNCgreen, TNC_sen, and DeltaTNC reduced the NRE (Figure 4c and Figure S14). The top three most important intrinsic factors of the PRE were TAC_sen, DeltaChl, and TNCgreen, with relative importance of 17.7%, 13.7%, and 12.7%, respectively (Figure 4b). The influence of TNCgreen and Chl_sen on the PRE was negative, resulting in a decrease in the PRE as TNCgreen and Chl_sen increased. TAC_sen and DeltaChl indicated a positive impact on the PRE compared with TNCgreen and Chl_sen (Figure 4d and Figure S15). The interaction effects between the influencing factors on the NRE and PRE are indicated using the summary plots of the SHAP interaction matrix values (Figure S16). The main effects are represented on the diagonal, while the interaction effects are depicted off the diagonal. The NRE and PRE were only marginally influenced by the factor interactions.

The quantitative relationship between the NRE (and PRE) and its influencing factors was estimated at an individual tree level (Figure 4e,f). Trees with the NRE (and PRE) of 50% were used for prediction. We summed up the SHAP values of all the influencing factors, and the actual prediction of the NRE (and PRE) was obtained based on the mean prediction (base value, E[f(x)]). Specifically, in the TreeSHAP model of the NRE, Chl_sen, EndDOY, Car_sen, TAC_sen, DeltaCar_per, TNC_sen, TNCgreen, and DeltaChl, with the values of 0.51 mg/g, 286 DOY, 0.76 mg/g, −0.15 mg/g, 54.09 mg/g, 12.93 mg/g, 11.15 mg/g, and 5.84 mg/g, respectively, together predicted the tree sample with the NRE of 50% (Figure 4e). In the TreeSHAP model of the PRE, TAC_sen, Chl_sen, DeltaTNC, PeakDOY, DeltaChl, TNCgreen, DeltaCar, TNC_sen, and Car_sen, with the values of 0.51 mg/g, 0.70 mg/g, 0.53 mg/g, 284 DOY, 5.51 mg/g, 11.76 mg/g, 0.95 mg/g, 12.29 mg/g, and 0.70 mg/g, respectively, together predicted the tree sample with the PRE of 50% (Figure 4f).

**Figure 5:**
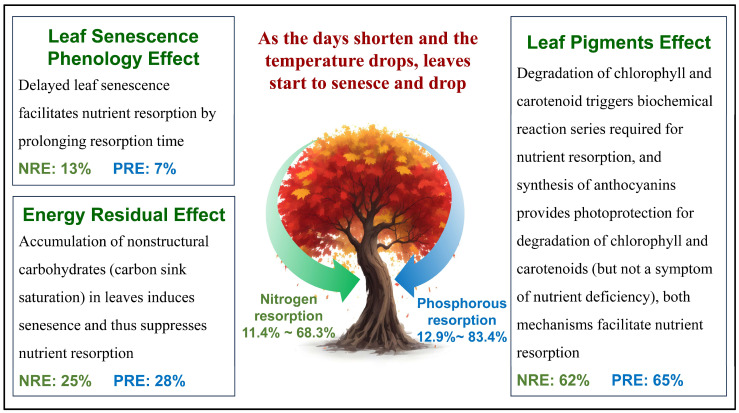
The three synergetic intrinsic ecological mechanisms of nitrogen and phosphorous resorption in temperate deciduous broad-leaved trees. The percentage (summed to 100%) is the relative importance of each ecological mechanism on nitrogen (N) or phosphorous (P) resorption efficiency across the 61 trees calculated by the random forest model.

Collectively, we proposed a diagram on the three intrinsic ecological mechanisms of NRE and PRE for our temperate deciduous broad-leaved trees (Figure 5).

## 3. Discussion

We quantitatively verified three new hypotheses on leaf NRE and PRE using an observational dataset of 61 tree individuals. Our results highlighted the roles of photosynthetic pigments’ degradation and anthocyanins’ biosynthesis on both N and P resorption. The intrinsic leaf factors of pigments, energy, and phenology together explained 85% and 60% of the variations in NRE and PRE, respectively. Pigments were the dominant factor influencing NRE and PRE variations across tree individuals. These novel findings extend the understanding of ecological mechanisms of N resorption to P resorption beyond the nutrient concentration-based variables and help improve the simulation and prediction of N and P resorption responses to climate change.

### 3.1. Leaf Senescence Phenology Hypothesis: Delayed Leaf Senescence Facilitates Nutrient Resorption

Significant variation in leaf senescence phenology was observed among the trees (Figure 1), and the leaf senescence date was positively correlated with NuRE (Figure S8), which supported the senescence phenology hypothesis. The positive effect of leaf senescence phenology on NuRE across tree species and individuals (Figure S8) [44,45] is in accordance with that within species [46,47]. During the extended time, more chlorophylls and carotenoids might be broken down, and more hormones and secondary metabolites could be synthesized [42], which will enhance NuRE (see the leaf pigments hypothesis for details). Interestingly, the correlations differed between NRE and PRE and changed with leaf-fall parameters (Figure S8). We noted that PRE was often more variable than NRE, both among species [2] and years [48], which might be due to the higher variability of leaf P components relative to N fractions [2]. The correlations of leaf-fall phenology with NRE were tighter than with PRE (Figure S8), while the best leaf-fall parameter for NuRE needs to be tested with more studies.

The senescence phenology hypothesis is of great significance for plant nutrient recycling in the context of global climate change. First, global warming is delaying the end date of the growing season and leaf senescence [22,49]. The extension of autumn prolongs the duration of nutrient resorption in leaves, potentially facilitating nutrient resorption. If the delay in leaf senescence persists in future as an adaptation to late-summer and autumn warming [22,50], our findings suggest that a warmer autumn is likely to result in higher NuRE. A more conserved nutrient strategy may help mitigate the nutrient deterioration due to the elevating atmospheric carbon dioxide concentration [51].

The species characteristics (such as frost tolerance) matter in interpreting and predicting the response of NuRE to climatic factors. The risk of frost damage may interrupt the nutrient resorption by sudden leaf shedding for frost-intolerant species. For example, in the present study, some *F*. *mandshurica* and *J*. *mandshurica* trees suffered from early frost, resulting in lower NuRE for the two tree species (Figure 3). If the cells are not abruptly killed by frost, these frost-intolerant tree species may resorb more nutrients [42]. In fact, the mean NRE and PRE for the two species in the year with early frost damage (2022) was much lower than that without early frost damage in 2017 [9] (NRE: 29.1% vs. 41.6%, PRE: 38.9% vs. 55.6%). Conversely, frost-resistant species (e.g., three tree species in this study (*A*. *mandshuricum*, *U*. *davidiana* var. *japonica*, and *Q*. *mongolica*) and *Populus tremula* [43]) are likely to benefit from warming autumns in nutrient resorption. Therefore, NuRE in response to early frost damage may be highly dependent on the frost tolerance of species. To better predict the response of NuRE to climate change, we need a new leaf senescence phenology model that considers the sensitivity of leaves to frost damage [52].

Among the leaf senescence phenology factors, we found that the end of leaf fall was the most important for predicting NuRE, while the date of peak of leaf fall was most relevant for PRE (Figure S7 and Figure 3), indicating that the best senescence phenology variable for NuRE is element-dependent. However, it is important to note that the marcescent leaf [44] of a few tree species (e.g., *Q*. *mongolica*) in some years poses a limitation when using both dates of the peak of leaf fall and end of leaf fall as predicting factors of NuRE. The widely used peak date of leaf coloration to represent leaf senescence phenology [53] also has limitations. This is because autumn leaf coloration may not occur in some species [24,29,54], and the early frost can damage leaves and induce sudden leaf abscission before coloration in some years (Figure S2) [55]; for example, in some individuals of the pinnately compound leaf species (*F*. *mandshurica* and *J*. *mandshurica*) in this study. These results suggested again that the species properties matter in detecting the best leaf-fall parameter for modeling NuRE.

### 3.2. Leaf Pigments Hypothesis: Degradation of Chlorophylls and Carotenoids and Synthesis of Anthocyanins Facilitate Nutrient Resorption

Quantitative measurements of leaf pigments provide a better understanding of the differences in NRE between red, yellow, and green leaves [36,38] through unveiling the nature of leaf color change. Our results, for the first time, demonstrated that the degradation of both chlorophylls and carotenoids enhanced not only NRE but also PRE (Figure S9). The minor amount of N in chlorophylls for green leaves (1.2~3.2%) and for senesced leaves (0.2~2.8%) in our studied trees indicated that the chlorophylls residual directly contributed little to NRE. However, chlorophyll degradation was related to the hydrolysis of proteins (such as Rubisco and chlorophyll-binding protein) and RNA [19], and biosynthesis of new products such as antioxidant tocopherol [30,56], both facilitating NRE and PRE. Remarkably, the degradation of carotenoids also promoted both N and P resorption (Figure S9), a new phenomenon seemingly at odds with the protective role of carotenoids from photooxidation and the comparable NRE between yellow and red leaves [29,57]. In fact, the photoprotective functions of carotenoids are weaker than anthocyanins [54], and degradation of carotenoids is also involved in degradation of N(P)-rich macromolecules and biosynthesis of secondary metabolites [31]. The changes in chlorophylls and carotenoids as simple indicators of the degree of degradation of chloroplasts (containing > 50% of total leaf N) determined the amount of chloroplast nutrients that remains at the end of senescence [2] and thus the NRE. The changes in chloroplast pigments had a less important role in PRE, as P fractions are more biochemically diverse than N fractions [2]. Comparison between chlorophylls and carotenoids showed that the breakdown of the former was more dramatic (Figure 2) [32,58] and important to NuRE (Figure 4). These results suggest that quantitative measurements of changes in chloroplast pigments (particularly chlorophylls) provided an integrated metric of the degree of leaf senescence, which are good indicators of leaf NuRE.

Interestingly, we observed that the role of anthocyanins in NuRE was more pronounced for P than N, although anthocyanins were only detected in some trees (Figure S10). Anthocyanins play a crucial role in maintaining photosynthesis before leaf senescence by providing photoprotection and scavenging free radicals [59], thereby enhancing energy availability for nutrient resorption and promoting leaf nutrient resorption [60]. It is important to acknowledge that there are numerous anthocyanin variants [26] and other red pigments [37], and thus there might be some quantification errors in representing anthocyanins as equivalents of cyanin-3-glucoside in our dataset. Note that we only detected anthocyanins in three species (the data were skewed around the zero values), which again suggested that species properties might impact the relationship between NuRE and anthocyanins. This highlighted the importance of selecting species in comparing the results among species and studies.

Despite this uncertainty in anthocyanins, we still found a significant positive correlation between NuRE and anthocyanin concentration in senesced leaves and excluded any indication of P and N deficiency by anthocyanins (Figure S11) c.f. [61]. Although two decades ago Steyn et al. [35] emphasized the symptom of P deficiency in many plants, there have been few quantifications of anthocyanins or leaf color types to indicate P resorption. Notably, we found that the importance of anthocyanins was stronger in PRE than NRE, which needs further studies to interpret. The chlorophyll degradation and synthesis of anthocyanins promoting N resorption in this study align with the significant difference in NRE between red and non-red (yellow and green) leaves, supporting the photoprotection of anthocyanins enhancing nutrient resorption [26,29,57,60]. Although we focused on N and P only, measuring changes in leaf pigments during leaf senescence is highly recommended to gain insight into the effect of leaf color on the resorption of other essential elements, such as sulfur, and metals.

Climate can have a significant impact on the changes in pigments in senescing leaves. Chlorophylls are more sensitive to temperatures [62] and light conditions [63] compared with carotenoids [31]. The decrease in autumn temperatures can disrupt the chloroplast membrane, leading to a more rapid degradation of chlorophylls than carotenoids. As a result, the reduction in carotenoids (46%) was notably lower than that of chlorophylls (71%) in our studied trees, causing most tree leaves to transition from green to yellow or brown. These results are in line with previous findings [32,42,58]. Furthermore, low temperatures and adequate radiation in autumn can stimulate the biosynthesis of anthocyanins in certain tree species [61]. In our study, most red-leafed species turned red after the first frost on September 20th, consistent with the previous observations [26]. Shading was found to delay the degradation of chlorophylls and suppress the biosynthesis of anthocyanins in understory leaves of a few individuals of *A*. *mandshuricum*, aligning with previous results [61,63]. These findings suggested that species properties will be important in explaining the inter-specific variation in NuRE. Manipulation experiments and multi-year measurements are particularly useful to accurately quantify the climatic sensitivity of leaf pigments and nutrient resorption.

### 3.3. Energy Residual Hypothesis: NSC Accumulation Suppresses Nutrient Resorption

We innovatively found that the NSC concentration before and after leaf senescence, rather than the change in NSC during senescence, suppressed N and P resorption, supporting the energy residual hypothesis (Figure S12). There were very few studies that explored the relationship between energy residual and NuRE in the literature. In a N addition experiment, NSC accumulation percentage during senescence was negatively corelated to the NRE and PRE in *Cunninghamia lanceolata* but not in *Liquidambar formosana* [64]. In physiology and biochemistry, soluble sugars are key substrates, not only as energy sources for the maintenance of cellular processes but also as signaling molecules during leaf senescence [65]. Numerous studies provided evidence on positive roles of sugars in leaf senescence at the physiological and genetic levels [65]. Accumulation of sugars can trigger leaf senescence by signaling a high availability of carbon relative to N in the aging leaves [41]. Indeed, our results suggested that soluble sugars and total NSCs in green and senesced leaves were negatively correlated to leaf senescence phenology across species and tree individuals (Table S4), strongly supporting that NSC accumulation can induce senescence [19]. Considering that NSC is a measure of carbon limitation (or energy balance) under various conditions [39,40], the negative correlation between NSCs and leaf senescence date also supports that the carbon sink limitation drives earlier autumn leaf senescence in temperate trees [66,67].

Soluble sugars and total NSCs before senescence were crucial intrinsic variables for simulated autumn leaf senescence and nutrient resorption, which were not considered in previous phenology and NuRE models. The effect of NSCs on NuRE also has great implications of climate change interactions. Under the context of increasing atmospheric CO_2_, there is evidence that the saturation of carbon sink in temperate trees has become a constraint of autumn leaf senescence delaying with warming temperature [67]. The decadal-weakened delaying senescence indicated that acclimation of phenology has enabled temperate plants to transcend a carbon sink saturation [50]. Unfortunately, the NSC concentration is difficult to retrieve from the historical monitoring of leaf phenology, hindering the direct testing of energy residual or carbon saturation hypothesis on both leaf senescence and nutrient resorption. In the field conditions, NSC concentration in green leaves can be related to many environmental factors [68], but the NSC concentration in senesced leaves was rarely measured except in a few litter decomposition studies [69]. Concomitant measurements of NSCs in field manipulation experiments (warming, rainfall reduction, fertilization, CO_2_ concentration elevation, etc.) in organs beyond leaves with nutrient resorption will be particularly helpful to deeply understand the variation in leaf phenology and nutrient resorption among years or decades, and across species [70].

### 3.4. Synthesis

Our research revealed that the three ecological mechanisms operated in coordination due to their interconnected nature. There were several lines of evidence on the possible synergistic effect of the three mechanisms of leaf nutrient resorption. Firstly, the positive effect of leaf-fall date on NuRE aligned with the negative impact of NSC concentration on NuRE, where higher NSC levels, especially soluble sugars, could accelerate early senescence (Figure S7) [65,71]. Secondly, the relationship between leaf-fall date and NuRE corresponded with the effects of changes in chloroplast pigments on NuRE (Figure S7). This was due to the prolonged biochemical reactions, including chloroplast pigment breakdown, resulting from delayed leaf abscission (not marcescence species) [2]. Thirdly, alterations in chloroplast pigments showed a negative correlation with NSC levels in green and senesced leaves (Figure S7), as elevated NSC concentrations could hasten leaf senescence. In a subtropical forest, a deciduous tree species that extended leaf senescence and accumulated anthocyanins demonstrated a synergistic enhancement in nutrient resorption compared to other deciduous species [45]. Our overall negative correlation between NSC in green leaves and final anthocyanins across all species did not contradict the concept that sugars induce anthocyanins biosynthesis in red-leaved species [61]. Finally, the TreeSHAP values highlighted the synergistic influence of the three mechanisms governing NuRE.

Although our study has novel findings, it is crucial to acknowledge certain limitations. Firstly, the modest sample size hindered our ability to discern the factors driving inter- and intra-specific variations. Secondly, we did not explore the effect of additional variables such as soil nutrient availability [5] and mycorrhizal associations [3] on NuRE. Further research to delineate potential synergistic or interactive effects of these three mechanisms with other factors is imperative. Thirdly, further research to delineate potential synergistic or interactive effects of these three mechanisms with other factors is imperative [10]. Long-term, multi-year data collection is indispensable for delving into our findings across individual, interannual, and decadal timeframes, enabling the development of a NuRE prediction model for climate change scenarios.

## 4. Materials and Methods

### 4.1. Site Description

The study was conducted at the Maoershan Forest Ecosystem Research Station (45°24′ N,127°40′ E), Heilongjiang Province, northeastern China. The site has a continental monsoon climate, with a mean annual temperature of 2.1 °C and mean precipitation of 726 mm from 2008 to 2019 [72]. The natural secondary forest was about 70 years in an elevation of approximately 400 m. The soils are classified as Alfisols in the United States Soil Taxonomy. The average C, N, and P concentrations in the topsoil (0–20 cm) were 66.74, 7.62, and 1.28 mg g^−1^, respectively. Previous studies found that this forest is only weakly N-limited [24,46].

### 4.2. Field and Laboratorial Measurements

We marked 61 individuals from 10 tree species based on autumn leaf color type and leaf phenology [24]. At least 3 healthy and mature individuals were selected from each species. For species with large intra-specific variation in autumn leaf color and abscission phenology, we selected 9 or 10 individuals to obtain a better representativeness (Table S1). Green leaf sampling: to represent the whole crown, we manually climbed each tree to cut a branch from the mid-to-upper canopy in early September (4–7 September 2022), just before leaf senescence started [73]. The early autumn was carefully determined for sampling time for mature leaves, by considering: (1) the N, P, and chlorophyll concentrations have not been entering the rapid decline phase based on the seasonality of leaf nutrients and chlorophylls [73], thus avoiding significant underestimation of nutrient concentration in mature leaves and, consequently, NuRE; (2) the NSC level has been past its depletion period during rapid summer growth [74], according to the carbon sink saturation hypothesis on leaf senescence phenology [67].

Senesced leaf sampling: From mid-September until mid-October, newly fallen leaves were collected on the ground as the senesced leaf sample according to the leaf phenology [9]. The fallen leaves well represented the whole crown because the fallen leaves were well mixed among vertical canopy layers. The percentage of leaves on the canopy that are colored and remaining is usually recorded by a visual estimation every two days, 10% of the leaves fallen (or 50% of the leaves on the crown changed color) as the start of leaf coloration (start of leaf fall), 50% of the leaves remaining as the peak of leaf fall, and 90% of the leaves fallen as the end of leaf fall [46]. We recorded the day of year (DOY) for start of leaf coloration, peak of leaf fall, and end of leaf fall changes for each tree.

All samples were brought back to the laboratory and transferred to a refrigerator as soon as possible. The samples of green leaves and senesced leaves were then measured for (1) leaf mass per area (LMA), (2) leaf pigments’ concentrations, and (3) NSC and nutrient concentrations. The detailed method description is listed in the Supplementary Materials.

### 4.3. Data Analysis

Nutrient resorption efficiency (NuRE) for each tree was calculated as follows:

(1) $$\mathrm{NuRE}\left(\%\right)=\left({\mathrm{Nu}}_{\mathrm{Gr}}-{\mathrm{Nu}}_{\mathrm{Sen}}\times \mathrm{MLCF}\right)/{\mathrm{Nu}}_{\mathrm{Gr}}\times 100\%$$

where Nu_Gr_ is the green leaf nutrient concentration (mg/g), Nu_Sen_ is the senesced leaf nutrient concentration, and MLCF is the species-specific leaf mass loss correction factor, which is previously measured at the same site [46,75].

To express change in leaf photosynthetic pigments and NSC during leaf senescence, we calculated the absolute (in mass concentration) and relative (in percentage) differences in chlorophylls, carotenoids, and NSC components before and after senescence (Table 1). Specifically, the decrease and its percentage in both total chlorophylls and carotenoids were used to express degradation. However, the changes in NSCs were either positive or negative, thus the change was not expressed as a consistent term of accumulation or depletion.

The differences in leaf chlorophylls, carotenoids, NSC, N, and P concentrations before and after leaf senescence among tree species were assessed using one-way analysis of variance (ANOVA) and Tukey’s method for multiple comparisons. The paired-samples *t*-test was used to analyze the changes in leaf chlorophylls, carotenoids, NSC, N, and P concentrations before and after leaf senescence. Additionally, Pearson correlation and simple linear regression were used to analyze the response of NuRE to each intrinsic parameter, including end of leaf fall, leaf chlorophylls, carotenoids, anthocyanins, NSCs, the degradation of chlorophylls and carotenoids, the change in NSCs during leaf senescence.

We found that there were no significant phylogenetic effects (*p* > 0.05) and soil nutrient availability on senesced leaf nutrients based on 46 woody species at the same site [24]; thus, we did not consider phylogeny and soil nutrient limitation in this study. Furthermore, none of the 10 species has N-fixing symbionts, excluding the contamination of N-fixing status on effects of intrinsic factors on NuRE and anthocyanins [36,38]. Although we found that the NRE was lower in arbuscular mycorrhizal than ectomycorrhiza fungi associated trees (40.2% versus 51.2%, *p* < 0.01), we did not consider this difference between the two mycorrhizal association types [5] because we sampled 5 species equally for each mycorrhizal type.

The random forest (RF) model and TreeExplainer-based SHAP (SHapley Additive exPlanations, TreeSHAP) method [76] were adopted to quantitatively estimate the factor contribution and interactive effects of the pigments, energy, and phenology variables for leaves (Table 1) on the NRE (or PRE). In the RF model, the predicting data were split into the training set and the test set (8:2). The grid search approach was used to tune model hyperparameters for the RF. Model validation was based on 10-fold cross-validation. Partial correlation was also used to quantify the effect of each intrinsic factor on NuRE, because this method excluded the influences of other factors. The TreeSHAP method was used to predict individual explanations (the influencing factors of the NRE (or PRE) for individual trees) and overall explanations (the influencing factors of the NRE (or PRE) in the whole region). The interactive effects between two variables and factor-positive or -negative effects on the NRE (or PRE) were directly estimated using the TreeSHAP. TreeSHAP was performed using the “shap” Python package. The RF models were implemented in the Scikit-learn package (version 0.24.2). The SHAP value unit is %. We employed the TreeSHAP algorithm to estimate the positive/negative and interacting effects of the predictors on leaf NuRE.

## 5. Conclusions

Our study validated three novel hypotheses that extend beyond the conventional explanatory variables based on nutrient concentration, which are typically used to regulate leaf NuRE. Specifically, the leaf senescence phenology hypothesis, leaf pigments hypothesis, and energy residual hypothesis were quantitatively confirmed to govern the intricate process of leaf nutrient resorption. The delayed leaf senescence, enhanced degradation of chlorophylls and carotenoids, and synthesis of anthocyanins promote both NRE and PRE. The significant impact of leaf color on NuRE was unveiled through direct measurements of the changes in leaf pigments. Furthermore, the accumulation of NSCs, particularly soluble sugars, was found to accelerate leaf senescence and simultaneously inhibit nutrient resorption. These findings represent a significant advancement in our understanding of N and P conservation in temperate trees and provide new insights into the complex interactions among plant nutrient resorption, carbon dynamics, and climate change.

## Figures and Tables

**Table 1:** The grouping of variables for verifying and quantifying the three intrinsic ecological mechanisms of leaf nutrient resorption efficiency.

Variable Group	Abbreviation	Definition
Phenology variables for leaf senescence phenology mechanism	StartDOY	Start of leaf fall
	PeakDOY	Peak of leaf fall
	EndDOY	End of leaf fall
Pigment variables for leaf pigments mechanism	Chl_sen	Chlorophyll in senesced leaves
	Car_sen	Carotenoids in senesced leaves
	TAC_sen	Anthocyanins in senesced leaves
	DeltaChl	Degradation of chlorophyll
	DeltaChl_per	Degradation percentage of chlorophyll
	DeltaCar	Degradation of carotenoids
	DeltaCar_per	Degradation percentage of carotenoids
Energy variables for energy residual mechanism	TNCgreen	Total nonstructural carbohydrates in green leaves
	TNC_sen	Total nonstructural carbohydrates in senesced leaves
	DeltaTNC	Change in total nonstructural carbohydrates (TNCs)
	DeltaTNC_per	Change percentage of TNCs

## Data Availability

Data are available from the Dryad Digital Repository: https://datadryad.org/stash/share/EB0GXmQY69DY0LkJxLP4X6mg4w2gf2L-UzONQJSDR5g, accessed on 15 February 2024.

## Supplementary Materials

The following supporting information can be downloaded at: https://www.mdpi.com/article/10.3390/plants13121659/s1. Method S1 Laboratorial analysis [46,77,78,79]. Table S1 Basic characteristics and number of individuals for the 10 tree species. Table S2 The relationship between nutrient resorption efficiency and change in nonstructural carbohydrates. Table S3 The relationship between nutrient resorption efficiency and ecophysiological parameters using species-mean data for the 10 tree species. Table S4 The correlation of nonstructural carbohydrates in green and senesced leaves with leaf senescence phenology and change in chlorophyll and carotenoids for the 10 tree species. Figure S1 Intercorrelations of the three parameters of leaf senescence phenology. Figure S2 Leaf color after senescence for the 10 species. Figure S3 Comparison of total chlorophyll concentration per leaf area before and after leaf senescence for the 10 species. Figure S4 Comparison of carotenoids concentration per leaf area before and after leaf senescence for the 10 tree species. Figure S5 Anthocyanins concentration in senesced leaves for the 10 tree species. Figure S6 Comparison of the concentration of total nonstructural carbohydrates per leaf area before and after leaf senescence for the 10 tree species. Figure S7 Correlation matrix of leaf nutrient resorption efficiency and its intrinsic factors and thermal time. Figure S8 Relationships of leaf nutrient resorption efficiency with leaf senescence phenological parameters. Figure S9 Relationships of leaf nutrient resorption efficiency with chlorophyll and carotenoids. Figure S10 Relationships of nutrient resorption efficiency with anthocyanins in senesced leaves. Figure S11 Relationships of green-leaf N and green-leaf P with anthocyanins. Figure S12 Relationship of leaf nutrient resorption efficiency with nonstructural carbohydrates in green leaves. Figure S13 Relationship of leaf nutrient resorption efficiency with nonstructural carbohydrates in senesced leaves. Figure S14 Partial correlations of intrinsic factors to nitrogen resorption efficiency. Figure S15 Partial correlations of intrinsic factors to phosphorous resorption efficiency. Figure S16 Summary plots of the SHAP interaction matrix values for the nutrient resorption efficiency on linear regression.
