# Supplementary material for: New Intrinsic Ecological Mechanisms of Leaf Nutrient Resorption in Temperate Deciduous Trees
Source: Plants (Basel). 2024 Jun 15;13(12):1659. doi: 10.3390/plants13121659 (PMC11207997; doi:10.3390/plants13121659)
Supplement: Supplementary file 1 [file plants-13-01659-s001.zip › plants-3032631-supplementary.pdf]

Supplementary Material

New intrinsic ecological mechanisms of leaf nutrient resorption in temperate deciduous trees

Method S1 Laboratorial analysis
Table S1 Basic characteristics and nutrient resorption efficiency for the 10 tree species
Table S2 Correlations of nutrient resorption efficiency with change of nonstructural carbohydrates
Table S3 Correlations of nutrient resorption efficiency with ecophysiological parameters using species-mean data for the 10 tree species
Table S4 Correlations of nonstructural carbohydrates in green and senesced leaves with leaf senescence phenology and change of chlorophylls and carotenoids for the 10 tree species
Figure S1 Intercorrelations of the three parameters of leaf senescence phenology
Figure S2 Leaf color after senescence for the 10 species
Figure S3 Comparison of total chlorophyll concentration per leaf area before and after leaf senescence for the 10 species
Figure S4 Comparison of carotenoids concentration per leaf area before and after leaf senescence for the 10 tree species
Figure S5 Anthocyanins concentration in senesced leaves for the 10 tree species
Figure S6 Comparison of the concentration of total nonstructural carbohydrates per leaf area before and after leaf senescence for the 10 tree species
Figure S7 Correlation matrix of leaf nutrient resorption efficiency and its intrinsic factors and thermal time
Figure S8 Correlations of leaf nutrient resorption efficiency with leaf senescence phenological parameters
Figure S9 Correlations of leaf nutrient resorption efficiency with chlorophylls and carotenoids
Figure S10 Correlations of nutrient resorption efficiency with anthocyanins in senesced leaves
Figure S11 Correlations of green-leaf N and green-leaf P with anthocyanins
Figure S12 Correlations of leaf nutrient resorption efficiency with nonstructural carbohydrates in green leaves
Figure S13 Correlations of leaf nutrient resorption efficiency with nonstructural carbohydrates in senesced leaves
Figure S14 Partial correlations of intrinsic factors to nitrogen resorption efficiency
Figure S15 Partial correlations of intrinsic factors to phosphorous resorption

efficiency

Figure S16 Summary plots of the SHAP interaction matrix values for the nutrient resorption efficiency on linear regression

Method S1 Laboratorial analysis

Leaf mass per area

Leaf mass per area was obtained for green and senesced leaves by measuring the fresh leaf area with 300-DPI scanned pictures and weighing the dry mass at 70°C for 48 hours.

Leaf pigments concentration

One hundred mg of fresh leaves was weighed and moved into centrifuge tubes with steel beads for chlorophylls and carotenoids, and 200 mg of fresh leaves frozen in liquid nitrogen was crushed in centrifuge tubes with glass beads for anthocyanins, before measurement. Chlorophylls and carotenoids were extracted using acetone- absolute alcohol- deionized water in a 4.5:4.5:1 ratio ultrasonic extraction in darkness, while anthocyanins were extracted by hydrochloric acid methanol-solution in a 2:98 ratio ultrasonic extraction in the dark, and the extracted solutions were balanced with corresponding pH buffers for measurement. Chlorophylls, carotenoids, and anthocyanins in the extracted solutions were measured using a spectrophotometer at different wavelengths (Eqs. 1-8). Leaf water content relative to fresh mass was also measured for converting fresh-mass-based pigments concentrations to dry-mass-based concentrations (%DM). Chlorophylls and carotenoids concentrations were calculated as follows:

$$\mathrm{Chla}(\mathrm{mg/g})=12.55A_{663.2}-2.79A_{646.8} \quad \text{(S1)}$$

$$\mathrm{Chlb}(\mathrm{mg/g})=21.50A_{646.8}-5.10A_{663.2} \quad \text{(S2)}$$

$$\mathrm{Total\ Chl}(\mathrm{mg/g})=7.15A_{663.2}+18.71A_{646.8} \quad \text{(S3)}$$

$$\mathrm{Carotenoids}(\mathrm{mg/g})=1000A_{470}-1.82\mathrm{Chla}-85.02\mathrm{Chlb}/198 \quad \text{(S4)}$$

where Chla is the concentration of chlorophyll a; Chlb is the concentration of chlorophyll b; total Chl is the total chlorophyll concentration; $A_{663.2}$ is the absorbance value of the sample at 663 nm; $A_{646.8}$ is the absorbance value of the sample at 647 nm; A_{470} is the absorbance value of the sample at 470 nm.

The anthocyanins concentration (only for senesced leaves) was measured with a pH differential method (Lee *et al.* 2005):

$$C_{ac}(\mathrm{mg/mL})=\Delta A\times M_w/(s\times L) \quad \text{(S5)}$$

where C is the total anthocyanins concentration; ΔA is the difference of absorptance at two pH values (Eq. 6); DF is dilution factor established in D; s indicates that the molar absorptivity of the final mixture is 26 900L/(mol/cm); Mw is the standard molecular weight of the final mixture of 449.2g/mol; and L indicates the optical range of the cuvette (1 cm).

$$\Delta A=\left[A_{510nm}(pH_{1.0})-A_{700nm}(pH_{1.0})\right]-\left[A_{510nm}(pH_{4.5})-A_{700nm}(pH_{4.5})\right] \quad \text{(S6)}$$

Then, we corrected the volumetric concentration (C_{ac}) with a standard curve using cyanidin-3-glucoside as the equivalent:

$$C_0=1.1974\times C_{ac}-0.0004 \quad \text{(S7)}$$

Volume concentrations were converted to anthocyanins concentration based

on fresh mass:

$$TAC = C_0 \times V_1 \times V_2 \times DF/m \quad (S8)$$

DF is the equilibrium dilution factor (Lee *et al.* 2005), V_1 and V_2 are the volume of the extractant and equilibrium constant volume. The water content of senesced leaves was used to convert the anthocyanins concentration based on fresh mass to that based on dry mass.

Leaf nonstructural carbohydrate concentration

The NSC was determined by an improved phenol-sulfuric acid method (Chow & Landhäusser 2004), which used enzymatic degradation of starch and thus avoided the overestimation of starch concentration caused by hydrolyzed hemicellulose and cellulose components by sulfuric acid. The NSC was defined as soluble sugars and starch, and the sum of the two referred to as total NSC (TNC), expressed as a percentage of dry mass (%DM) (Zhang *et al.* 2014). The concentrations of soluble sugar and starch were determined based on the sucrose standard curve.

Leaf nutrient concentration

N and P for green and senesced leaves were digested with concentrated sulfuric acid and hydrogen peroxide, and then measured using a continuous flow analyzer (AA3, Bran+luebbe, Germany) (Wang *et al.* 2022).

References

Chow, P.S. & Landhäusser, S.M. (2004). A method for routine measurements of total sugar and starch content in woody plant tissues. *Tree Physiology*, 24, 1129-1136.

Lee, J., Durst, R.W. & Wrolstad, R.E. (2005). Determination of total monomeric anthocyanin pigment content of fruit juices, beverages, natural colorants, and wines by the pH differential method: Collaborative study. *Journal of Aoac International*, 88, 1269-1278.

Wang, X.C., Song, H.M., Liu, F., Quan, X.K. & Wang, C.K. (2022). Timing of leaf fall and changes in litter nutrient concentration compromise estimates of nutrient fluxes and nutrient resorption efficiency. *Forest Ecology and Management*, 513, 120188.

Zhang, H.Y., Wang, C.K. & Wang, X.C. (2014). Spatial variations in non-structural carbohydrates in stems of twelve temperate tree species. *Trees-Structure and Function*, 28, 77-89.

Table S1 Basic characteristics and nutrient resorption efficiency for the 10 tree species. AM: arbuscular mycorrhizal fungi, EcM: ectomycorrhiza fungi. NRE: nitrogen resorption efficiency; PRE: phosphorous resorption efficiency.

Species	Mycorrhizal type	Leaf form	Number of tree individual	Mass loss correction factor	NRE (%)	PRE (%)
Populus koreana	EcM	Simple leaf	3	0.798	41.2 ± 1.97	35.3 ± 3.23
Juglans mandshurica	AM	Pinnately compound leaf	9	0.896	27.1 ± 3.48	37.4 ± 4.40
Syinga reticulata var. *mandshurica*	AM	Simple leaf	3	0.893	35.4 ± 1.85	39.2 ± 6.19
Fraxinus mandshurica	AM	Pinnately compound leaf	9	0.888	31.1 ± 2.31	40.4 ± 4.03
Betula costata	EcM	Simple leaf	3	0.869	51.1 ± 4.32	27.2 ± 3.97
Ulmus davidiana var. *japonica*	EcM	Simple leaf	10	0.892	46.1 ± 2.62	51.5 ± 3.41
Acer pictum subsp. *mono*	AM	Simple leaf	3	0.911	55.4 ± 1.97	50.8 ± 4.03
Betula platyphylla	EcM	Simple leaf	3	0.847	52.4 ± 4.14	34.8 ± 6.35
Acer mandshuricum	AM	Simple leaf	9	0.876	58.8 ± 2.78	69.9 ± 3.39
Quercus mongolica	EcM	Simple leaf	9	0.850	60.0 ± 2.15	43.2 ± 2.53
Mean ± SE				0.872 ± 0.01	45.3 ± 4.1	45.8 ± 1.96

Table S2 Correlations of nutrient resorption efficiency with change of nonstructural carbohydrates. DeltaTNC – change of total nonstructural carbohydrates, DeltaSSugars – change of soluble sugars, DeltaStarch – change of starch, DeltaTNC_per – change percentage of total nonstructural carbohydrates, DeltaSSugars_per – change percentage of soluble sugars, DeltaStarch – change percentage of starch. The same below.

Variable	NRE	PRE
DeltaTNC	–0.217NS	0.046NS
DeltaSSugars	–0.223NS	0.078NS
DeltaStarch	0.02NS	–0.127NS
DeltaTNC_per	–0.083NS	0.131NS
DeltaSSugars_per	–0.070NS	0.176NS
DeltaStarch_per	0.094NS	–0.070NS

Table S3 Correlation of nutrient resorption efficiency with ecophysiological parameters using species-mean data for the 10 tree species

Variable	NRE		PRE	
	R (5 tree species)	*R* (10 tree species)	*R* (5 tree species)	*R* (10 tree species)
StartDOY	0.967**	0.736*	0.720 NS	0.602 NS
PeakDOY	0.990**	0.823**	0.629 NS	0.459 NS
EndDOY	0.973**	0.826**	0.479 NS	0.273 NS
Chl_sen	-0.884*	-0.814**	-0.458 NS	-0.279 NS
Car_sen	-0.963**	-0.646*	-0.523 NS	-0.015 NS
TAC_sen	0.552 NS	0.415 NS	0.963**	0.909**
TNCgreen	-0.810 NS	-0.718*	-0.774 NS	-0.644*
SSugarsgreen	-0.749 NS	-0.672*	-0.703 NS	-0.667*
Starchgreen	-0.658 NS	-0.566NS	-0.720 NS	-0.251 NS
TNC_sen	-0.743 NS	-0.739*	-0.737 NS	-0.252 NS
SSugars_sen	-0.711 NS	-0.729*	-0.624 NS	-0.182 NS
Starch_sen	-0.427 NS	-0.523 NS	-0.901*	-0.476 NS
DeltaChl	0.793 NS	0.692*	0.891*	0.705*
DeltaCar	0.592 NS	0.523 NS	0.799 NS	0.596 NS
DeltaTNC	-0.039 NS	-0.302 NS	-0.078 NS	0.279 NS
DeltaSSugars	-0.049 NS	-0.253 NS	0.012 NS	-0.182 NS
DeltaStarch	0.088 NS	-0.166 NS	-0.576 NS	-0.476 NS
DeltaChl_per	0.958*	0.877**	0.600 NS	0.518 NS
DeltaCar_per	0.860 NS	0.728*	0.743NS	0.381 NS

Variable	NRE		PRE	
	R (5 tree species)	*R* (10 tree species)	*R* (5 tree species)	*R* (10 tree species)
DeltaTNC_per	0.272 NS	-0.152 NS	0.261NS	0.408 NS
DeltaSSugars_per	0.285 NS	-0.089NS	0.410 NS	0.518 NS
DeltaStarch_per	0.215 NS	0.070 NS	-0.413 NS	-0.195 NS

StartDOY – start of leaf coloration (or leaf fall), PeakDOY – peak of leaf fall, EndDOY – end of leaf fall, Chl_sen – chlorophylls in senesced leaves, Car_sen – carotenoids in senesced leaves, TAC_sen – anthocyanins in senesced leaves, TNCgreen – total nonstructural carbohydrates in green leaves, SSugarsgreen – soluble sugars in green leaves, Starchgreen – starch in green leaves, TNC_sen – total nonstructural carbohydrates in senesced leaves, SSugars_sen – soluble sugars in senesced leaves, Starch_sen – starch in senesced leaves, DeltaChl – degradation of chlorophylls, DeltaCar – degradation of carotenoids, DeltaTNC – change of total nonstructural carbohydrates, DeltaSSugars – change of soluble sugars, DeltaStarch – change of starch, DeltaChl_per – degradation percentage of chlorophylls, DeltaCar_per – degradation percentage of carotenoids, DeltaTNC – change of total nonstructural carbohydrates, DeltaSSugars – change of soluble sugars, DeltaStarch – change of starch. DeltaTNC_per – change percentage of total nonstructural carbohydrates, DeltaSSugars_per – change percentage of soluble sugars, DeltaStarch_per – change percentage of starch. The same below. **, * indicate significant at the 0.01 and 0.05 levels, respectively

Table S4 Correlations of nonstructural carbohydrates in green and senesced leaves with leaf senescence phenology and change of chlorophylls and carotenoids for the 10 tree species

Variable	SSugarsgreen	Starchgreen	TNCgreen	SSugars_sen	Starch_sen	TNC_sen
StartDOY	-0.554**	-0.122 NS	-0.548**	-0.160 NS	-0.240 NS	-0.200 NS
PeakDOY	-0.563**	-0.136 NS	-0.550**	-0.319*	-0.246 NS	-0.346**
EndDOY	-0.503**	-0.189 NS	-0.508**	-0.338**	-0.121 NS	-0.385**
Chl_sen	0.311*	0.281*	0.352**	0.599**	0.220 NS	0.600**
Car_sen	0.240NS	0.086 NS	0.239 NS	0.427**	0.118 NS	0.420**
DeltaChl	-0.610**	-0.217 NS	-0.616**	-0.355**	-0.363**	-0.403**
DeltaCar	-0.484**	-0.020 NS	-0.453**	-0.143 NS	-0.280*	-0.189NS
DeltaChl_per	-0.410**	-0.282*	-0.446**	-0.514**	-0.233 NS	-0.524**
DeltaCar_per	-0.388**	0.002 NS	-0.357**	-0.239 NS	-0.206 NS	-0.263*

**, * indicate significant at the 0.01 and 0.05 levels, respectively

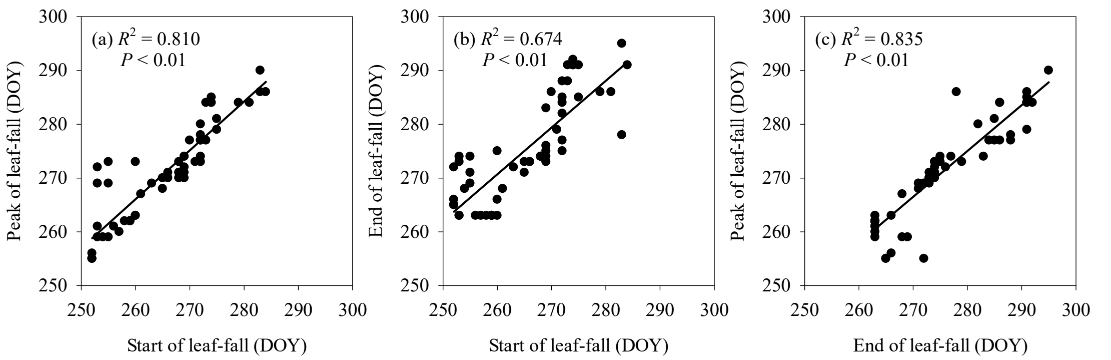

Figure S1 Intercorrelations of the three parameters of leaf senescence phenology

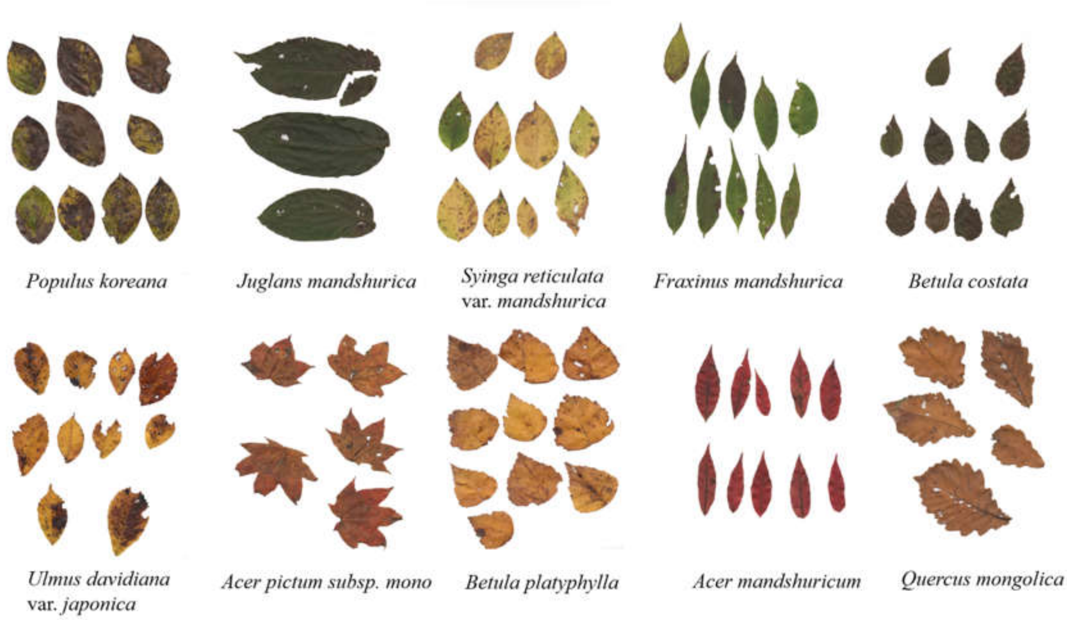

Figure S2 Leaf color after senescence for the 10 species. Senesced leaves of *A. mandshuricum*, some *Acer pictum* subsp. *mono*, and some *U. davidiana* var. *japonica* were red or orange-red; *Q. mongolica*, *B. costata*, some *U. davidiana* var. *japonica* had brown senesced leaves; *B. platyphylla*, some *A. pictum* subsp. *mono*, and some *U. davidiana* var. *japonica* had orange-yellow leaves; *S. reticulata* var. *mandshurica* and some *J. mandshurica* had yellow-green leaves. Specifically, due to a sudden drop in temperature and early frost on September 20th, most *F. mandshurica* and *J. mandshurica* trees were unable to change color (remained green) when shedding.

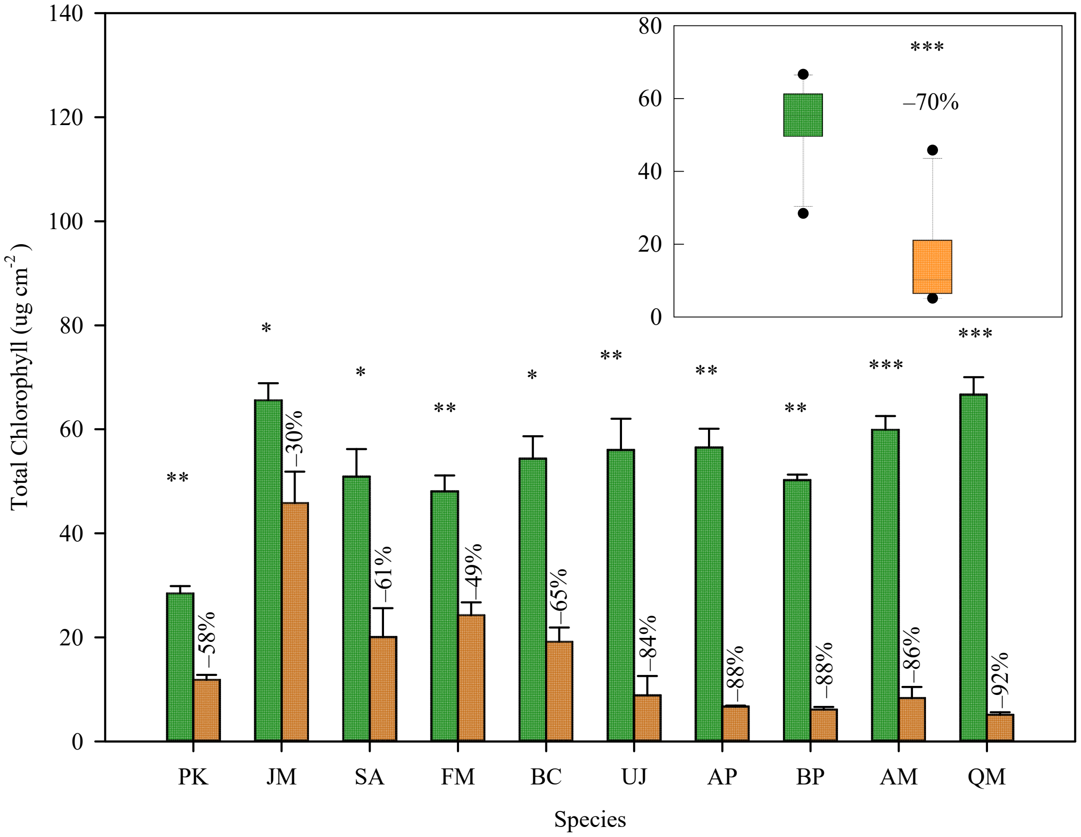

Figure S3 Comparison of total chlorophyll concentration per leaf area before and after leaf senescence for the 10 species. Species abbreviations: *Populus koreana* (PK), *Juglans mandshurica* (JM), *Syinga reticulata* var. *mandshurica* (SA), *Fraxinus mandshurica* (FM), *Betula costata* (BC), *Ulmus davidiana* var. *japonica* (UJ), *Acer pictum* subsp. *mono* (AM), *Betula platyphylla* (BP), *Acer mandshuricum* (AM), and *Quercus mongolica* (QM). The green box indicates green leaves and brown indicates senesced leaves, and the error bar is standard error. The change percentage is shown on each histogram. The insert figure is the mean of the 10 species. The same below. ***, **, * indicate significant at the 0.001, 0.01, and 0.05 levels, respectively

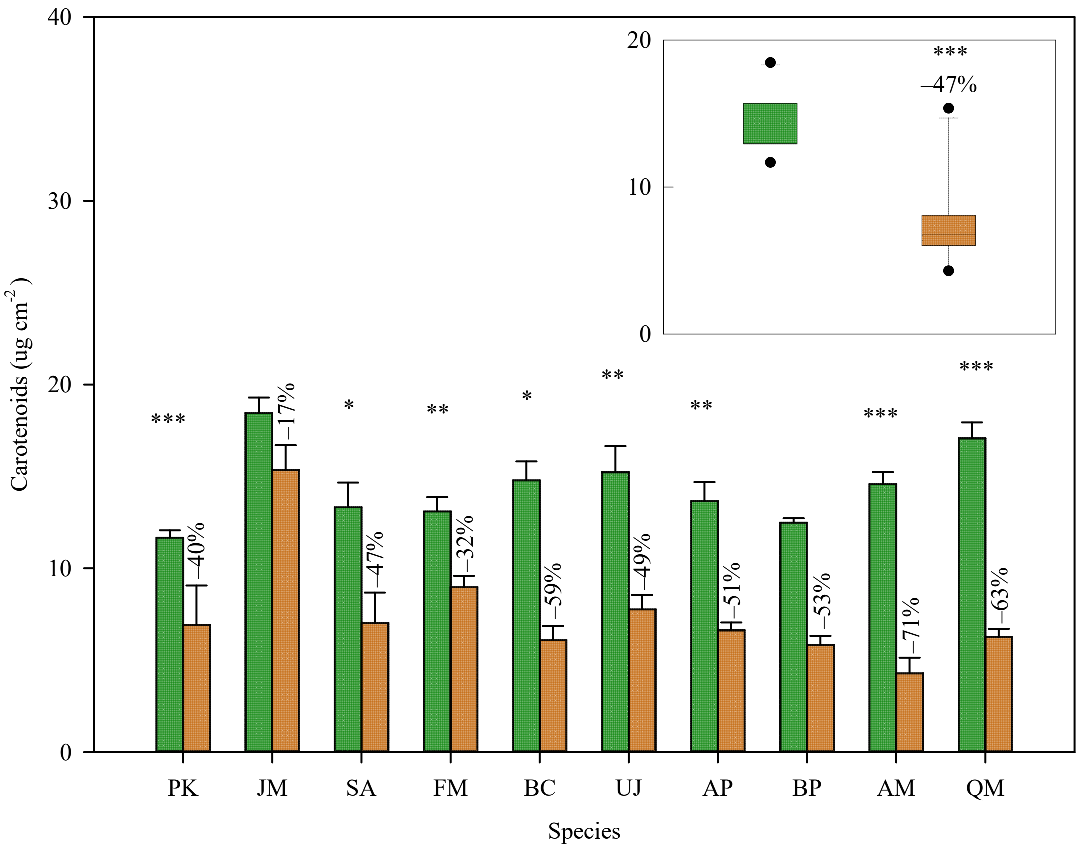

Figure S4 Comparison of carotenoids concentration per leaf area before and after leaf senescence for the 10 species. The green box indicates green leaves and brown indicates senesced leaves, and the error bar is standard error. The change percentage is shown on each histogram. The insert figure is the mean of the 10 species. Species abbreviations are listed in Figure S3. ***, **, * indicate significant at the 0.001, 0.01, and 0.05 levels, respectively

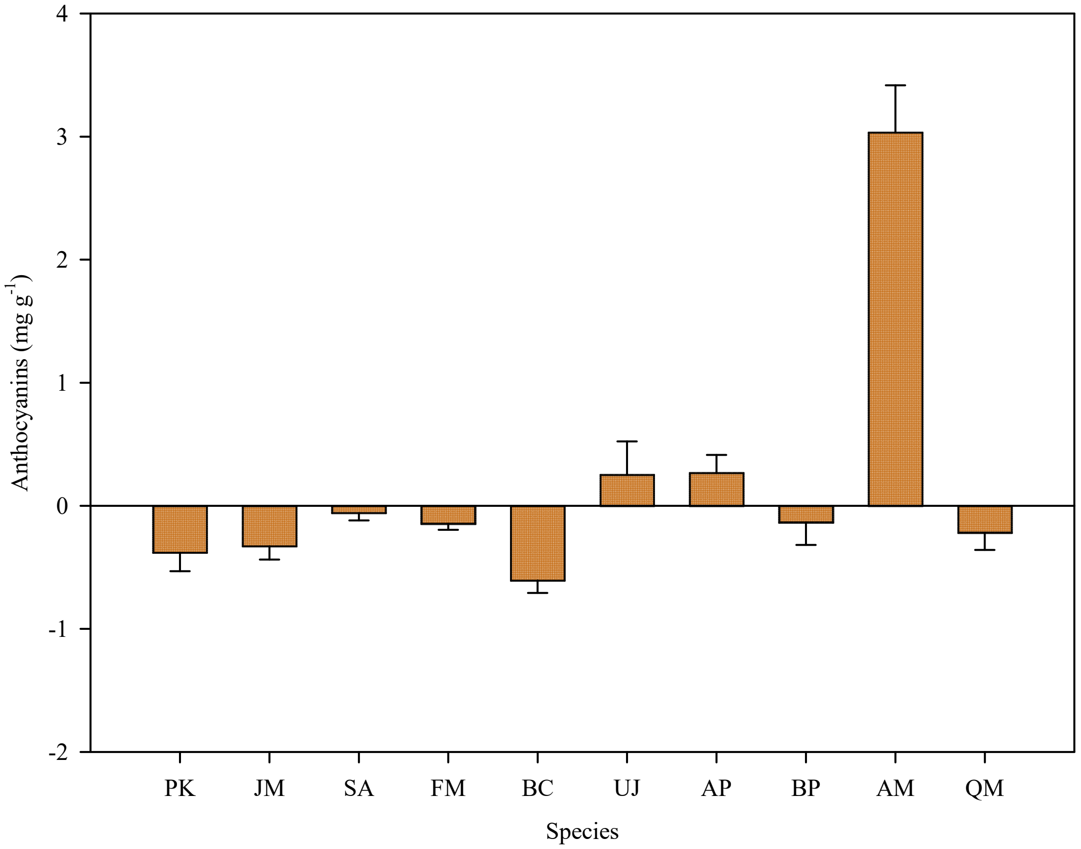

Figure S5 Anthocyanins concentration in senesced leaves for the 10 tree species

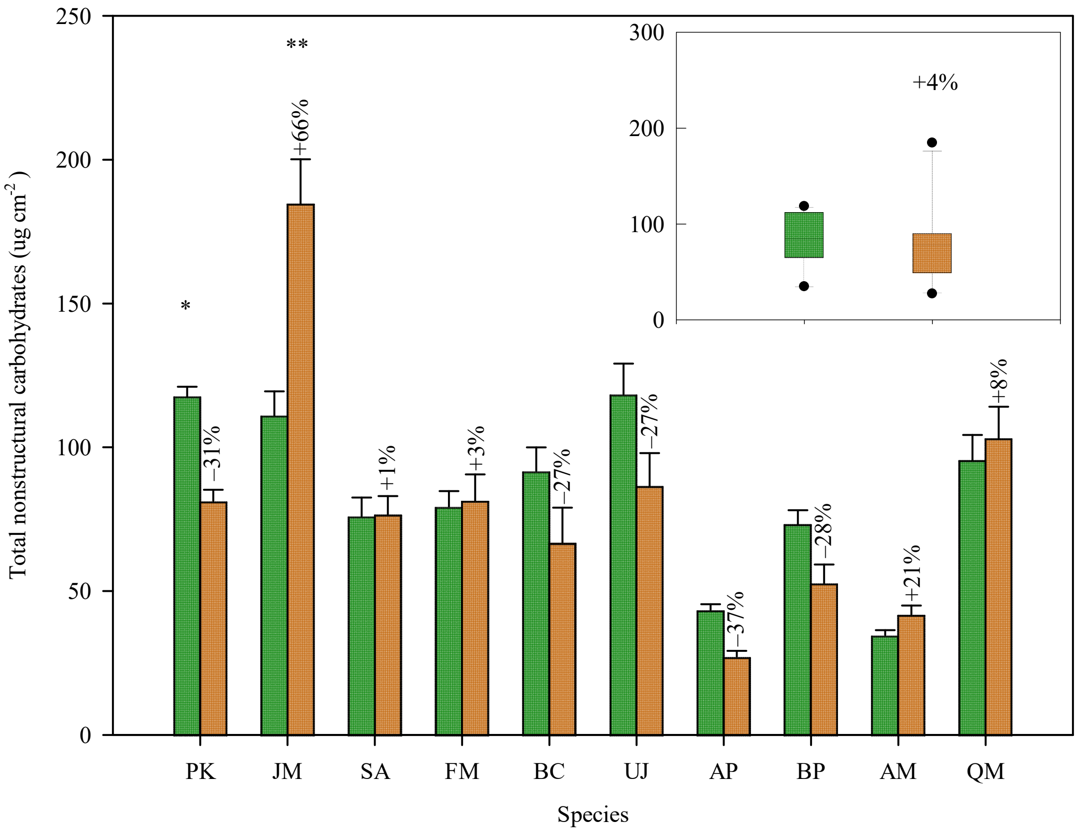

Figure S6 Comparison of the concentration of total nonstructural carbohydrates per leaf area before and after leaf senescence for the 10 tree species. The green box indicates green leaves and brown indicates senesced leaves, and the error bar is standard error. The change percentage is shown on each histogram. The insert figure is the mean of the 10 species. Species abbreviations are listed in Figure S3. **, * indicate significant at the 0.01 and 0.05 levels, respectively

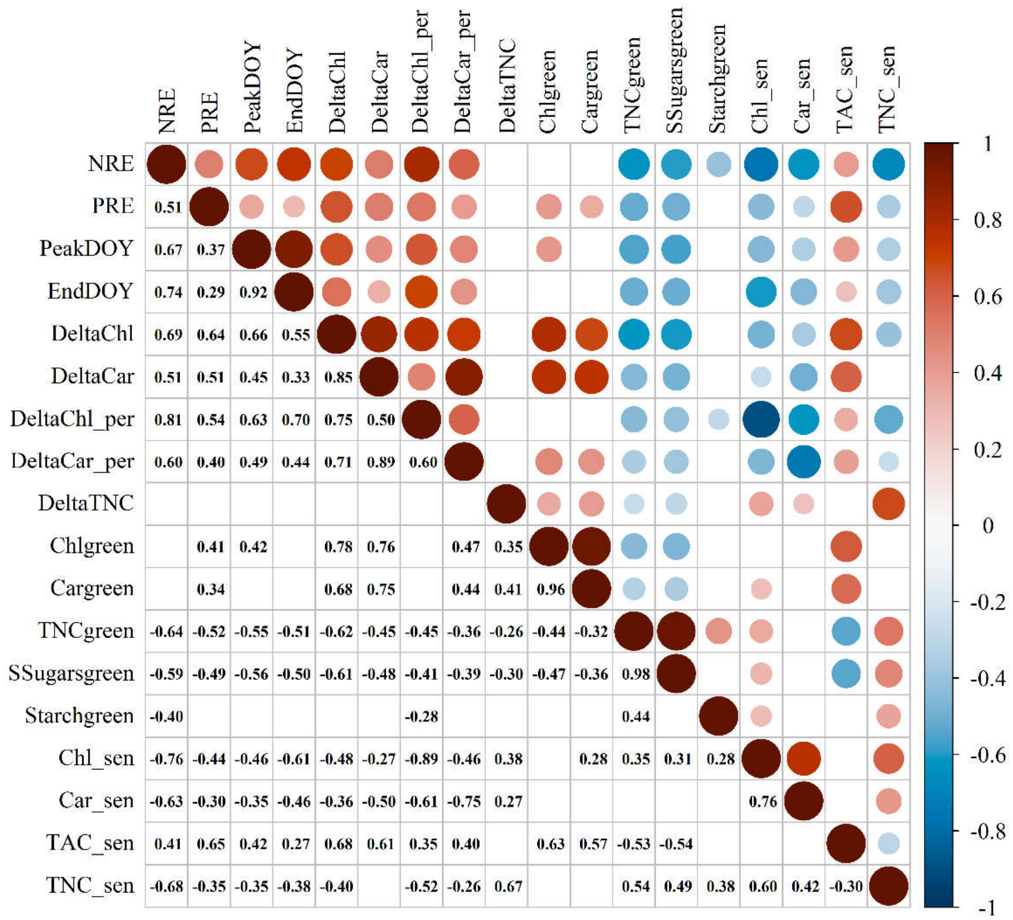

Figure S7 Correlation matrix of leaf nutrient resorption efficiency and its intrinsic factors and thermal time. PeakDOY – peak of leaf fall, EndDOY – end of leaf fall, DeltaChl – degradation of chlorophylls, DeltaCar – degradation of carotenoids, DeltaChl_per – degradation percentage of chlorophylls, DeltaCar_per – degradation percentage of carotenoids, DeltaTNC – change of total nonstructural carbohydrates, Chl_ green – chlorophylls in green leaves, Car_ green – carotenoids in green leaves, TNC_green – total nonstructural carbohydrates in green leaves, SSugars_green – soluble sugars in green leaves, Starch_green – starch in green leaves, Chl_sen – chlorophylls in senesced leaves, Car_sen – carotenoids in senesced leaves, TAC_sen – anthocyanins in senesced leaves, TNCsen – total nonstructural carbohydrates in senesced leaves. The same below.

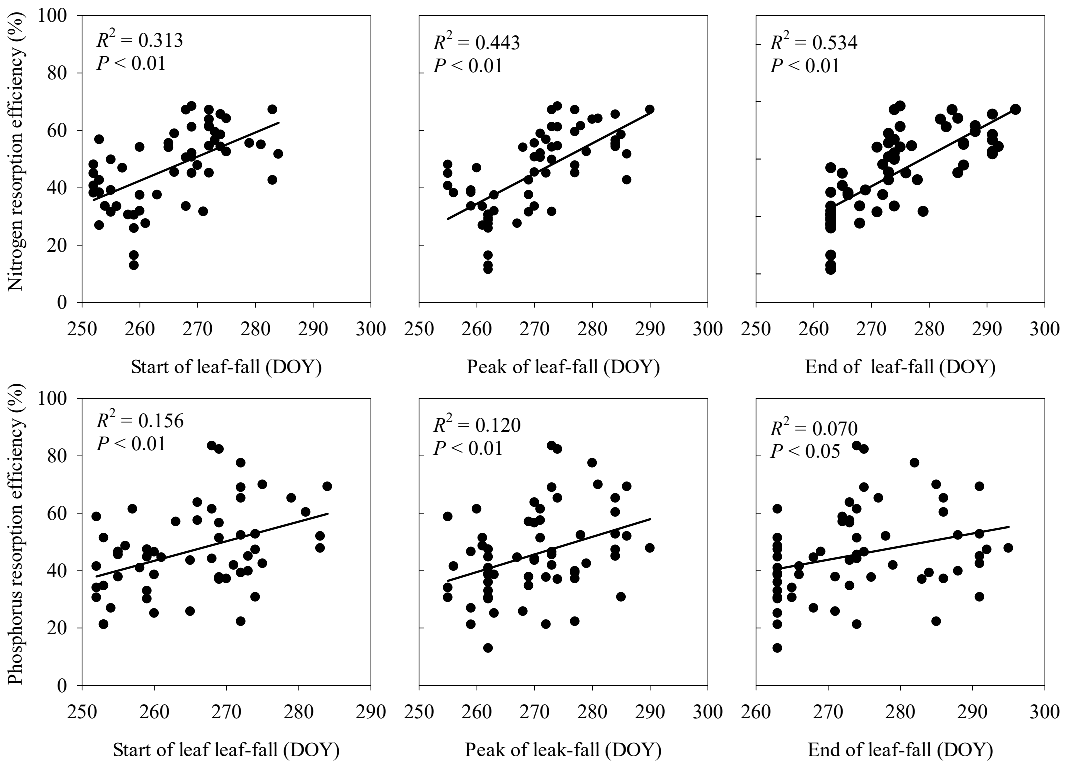

Figure S8 Correlations of nutrient resorption efficiency with leaf senescence phenological parameters

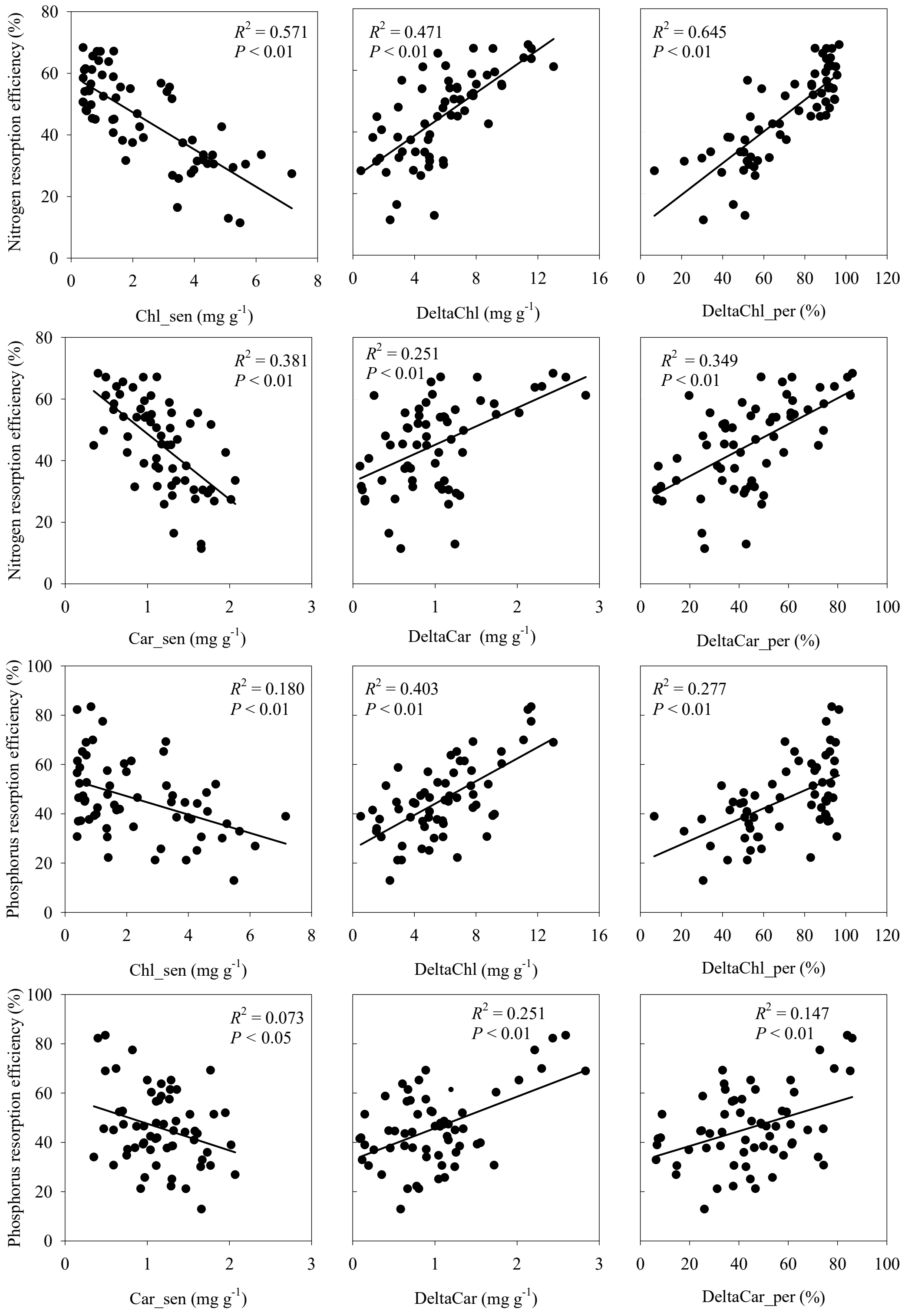

Figure S9 Correlations of leaf nutrient resorption efficiency with chlorophylls and carotenoids

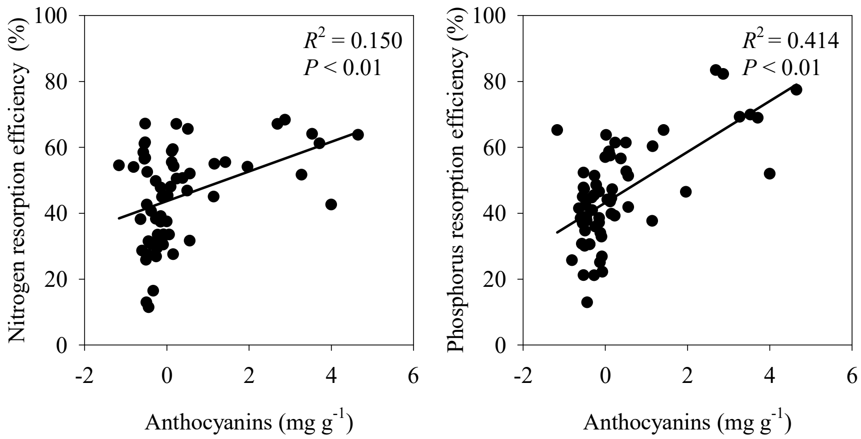

Figure S10 Correlations of nutrient resorption efficiency with anthocyanins in senesced leaves

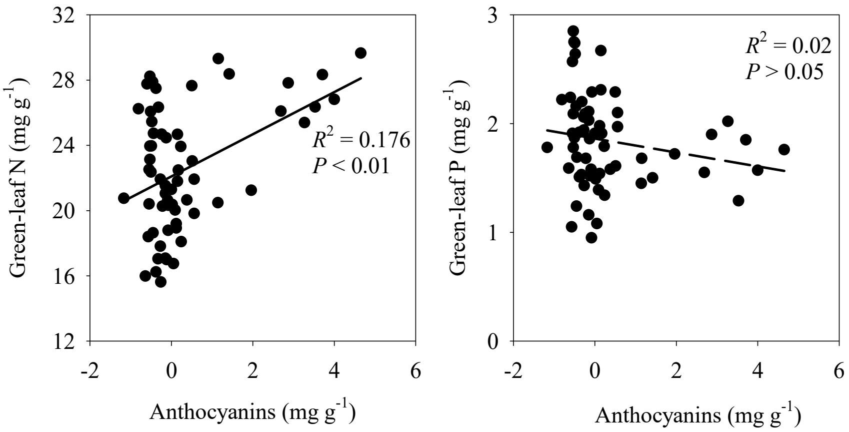

Figure S11 Correlations of green-leaf N and green-leaf P with anthocyanins. The solid line indicates a significant Correlation, while the dashed line indicates an insignificant one.

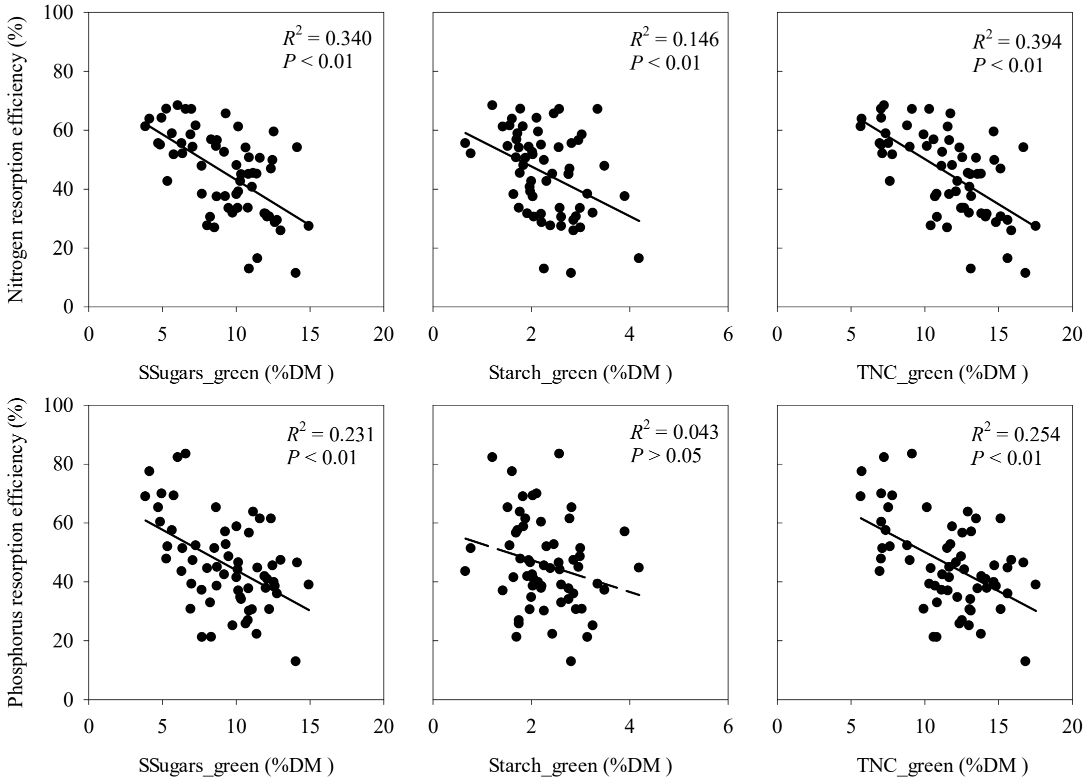

Figure S12 Correlations of leaf nutrient resorption efficiency with nonstructural carbohydrates in green leaves. The solid line indicates a significant Correlation, while the dashed line indicates an insignificant one.

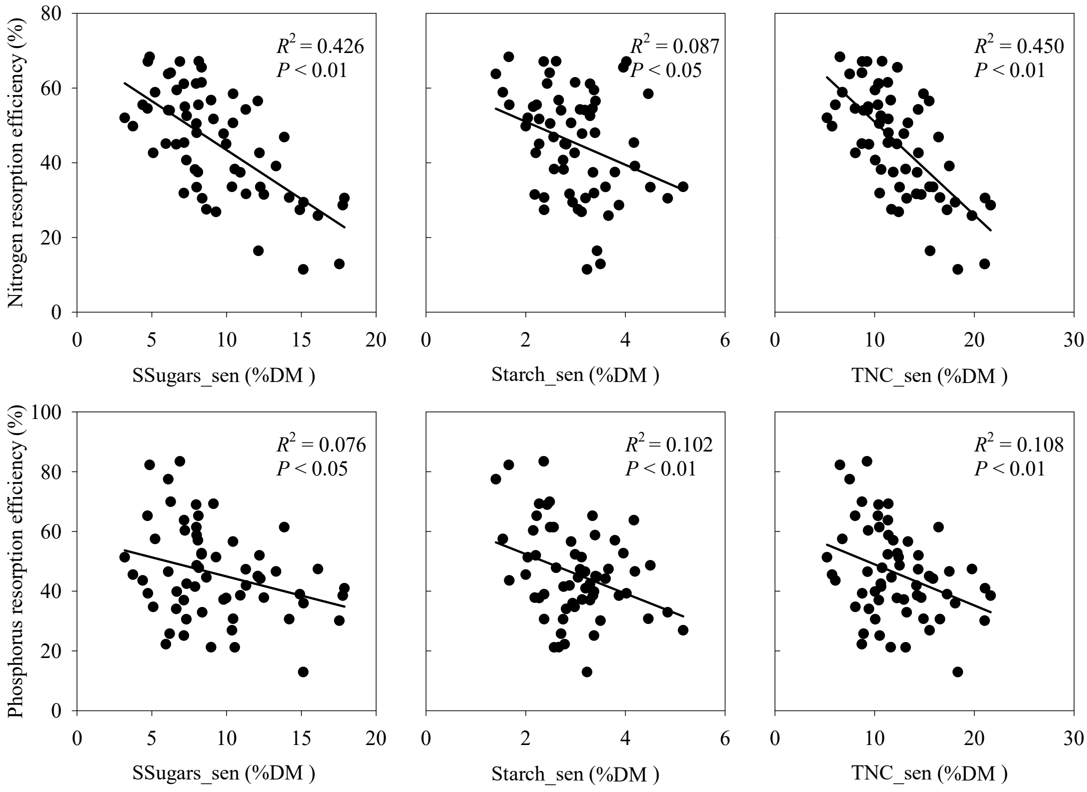

Figure S13 Correlations of leaf nutrient resorption efficiency with nonstructural carbohydrates in senesced leaves

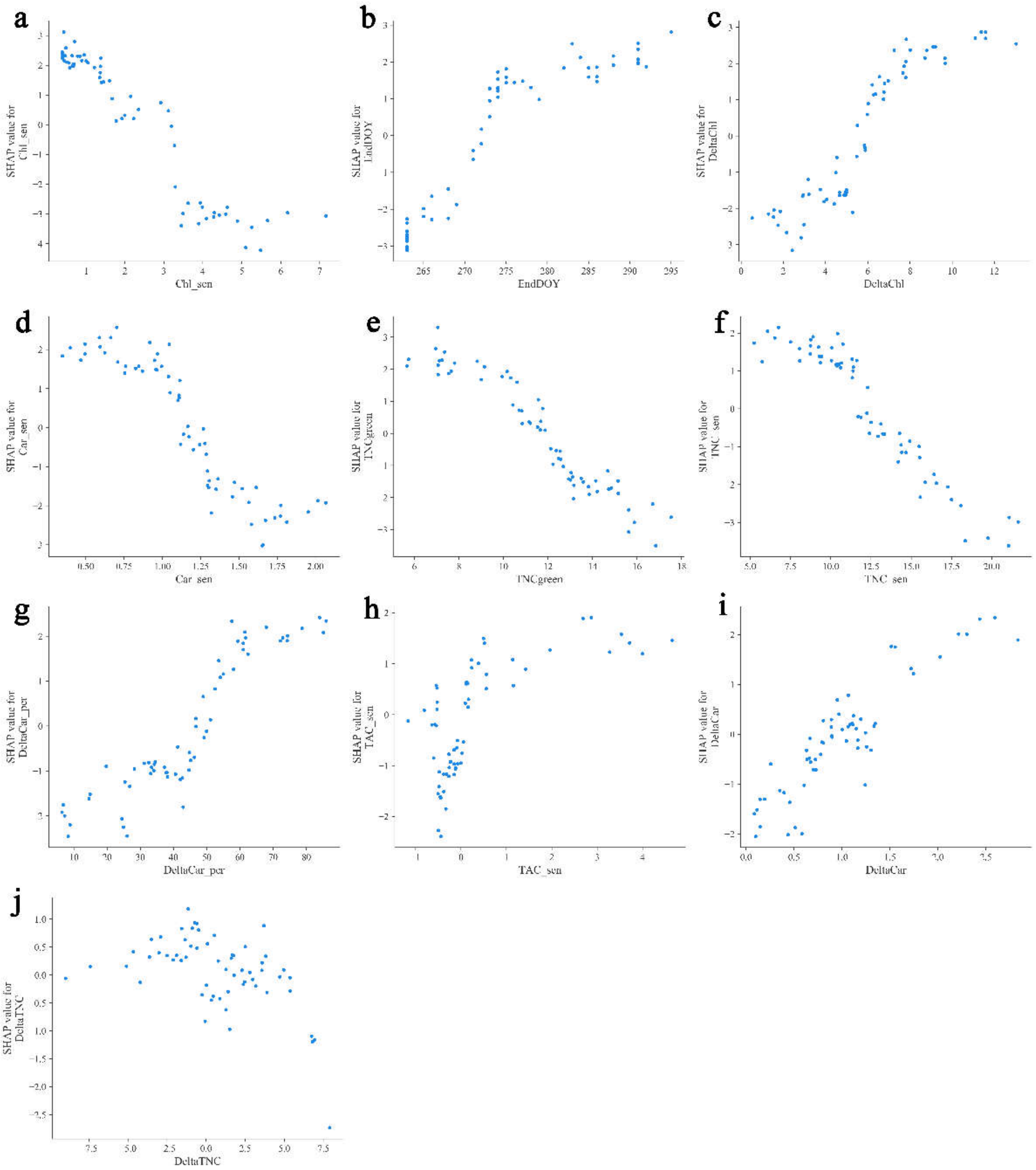

Figure S14 Partial correlations of intrinsic factors to nitrogen resorption efficiency

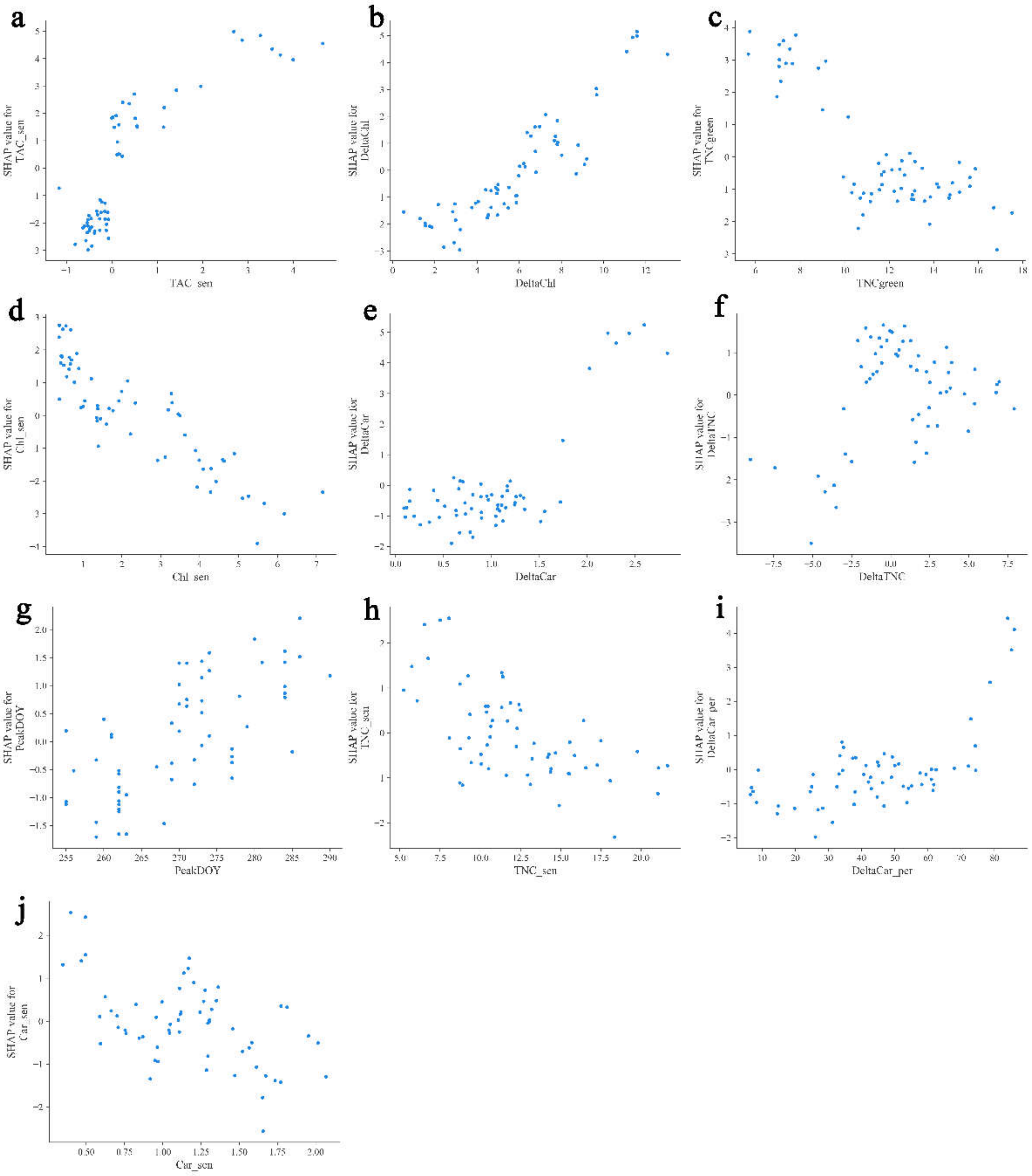

Figure S15 Partial correlations of intrinsic factors to phosphorous resorption efficiency

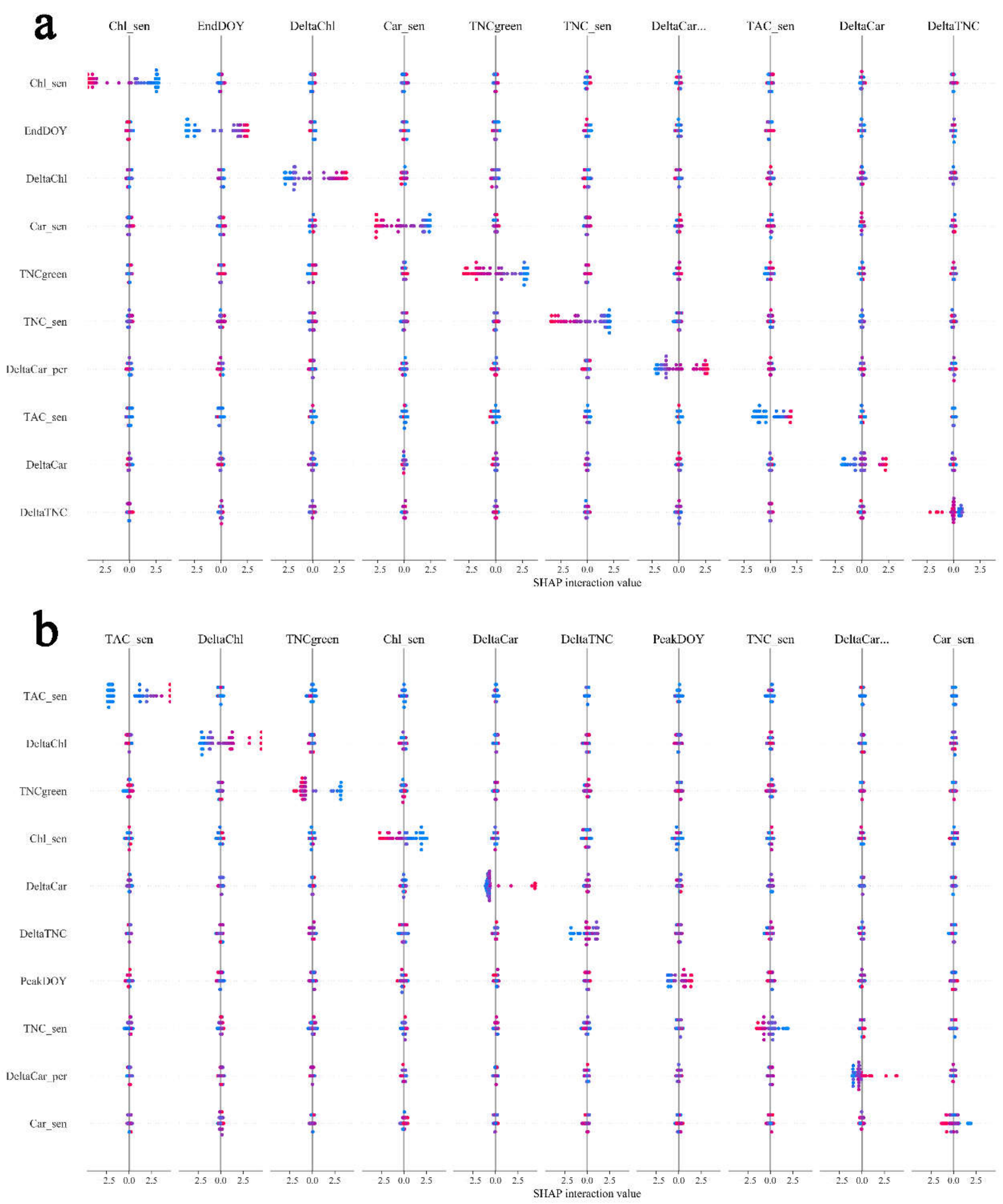

Figure S16 Summary plots of the SHAP interaction matrix values for the nutrient resorption efficiency. (a) NRE and (b) PRE.
